# A novel leishmanial copper P-type ATPase plays a vital role in parasite infection and intracellular survival

**DOI:** 10.1016/j.jbc.2021.101539

**Published:** 2021-12-25

**Authors:** Rupam Paul, Sourav Banerjee, Samarpita Sen, Pratiksha Dubey, Saptarshi Maji, Anand K. Bachhawat, Rupak Datta, Arnab Gupta

**Affiliations:** 1 Department of Biological Sciences, Indian Institute of Science Education and Research Kolkata, Mohanpur, West Bengal, India; 2 Department of Biological Sciences, Indian Institute of Science Education and Research Mohali, Sahibzada Ajit Singh Nagar, Punjab, India

**Keywords:** ATP7, *Leishmania*, copper, Cu-ATPase, lysosome, ATP7A, host–pathogen interaction, *ATOX1*, antioxidant protein 1, cDNA, complementary DNA, CTR1, Cu transporter 1, CTR2, Cu transporter 2, Cu, copper, DAPI, 4′,6-diamidino-2-phenylindole, DPBS, Dulbecco's PBS, DST, Department of Science and Technology, HM, heavy metal, ICP–OES, inductively coupled plasma optical emission spectrometry, IISER, Indian institute of Science Education and Research, Lamp1/2, lysosome-associated membrane protein 1/2, LmATP7-Het, *LmATP7* heterozygous deletion, LmATP7-OE, *LmATP7* overexpressing, PDB, Protein Data Bank, p.i., postinfection, qRT–PCR, quantitative RT–PCR, SD, synthetic defined, SD-Ura, SD medium without uracil, TGN, *trans*-Golgi network

## Abstract

Copper (Cu) is essential for all life forms; however, in excess, it becomes toxic. Toxic properties of Cu are known to be utilized by host species against various pathogenic invasions. *Leishmania*, in both free-living and intracellular forms, exhibits appreciable tolerance toward Cu stress. While determining the mechanism of Cu-stress evasion employed by *Leishmania*, we identified and characterized a hitherto unknown Cu-ATPase in *Leishmania major* and established its role in parasite survival in host macrophages. This novel *L. major* Cu-ATPase, LmATP7, exhibits homology with its orthologs at multiple motifs. In promastigotes, LmATP7 primarily localized at the plasma membrane. We also show that *LmATP7* exhibits Cu-dependent expression patterns and complements Cu transport in a Cu-ATPase-deficient yeast strain. Promastigotes overexpressing LmATP7 exhibited higher survival upon Cu stress, indicating efficacious Cu export compared with Wt and heterozygous *LmATP7* knockout parasites. We further explored macrophage–*Leishmania* interactions with respect to Cu stress. We found that *Leishmania* infection triggers upregulation of major mammalian Cu exporter, ATP7A, in macrophages, and trafficking of ATP7A from the *trans*-Golgi network to endolysosomes in macrophages harboring amastigotes. Simultaneously, in *Leishmania*, we observed a multifold increase in *LmATP7* transcripts as the promastigote becomes established in macrophages and morphs to the amastigote form. Finally, overexpressing *LmATP7* in parasites increases amastigote survivability within macrophages, whereas knocking it down reduces survivability drastically. Mice injected in their footpads with an LmATP7-overexpressing strain showed significantly larger lesions and higher amastigote loads as compared with controls and knockouts. These data establish the role of LmATP7 in parasite infectivity and intramacrophagic survivability.

*Leishmania* is a digenetic protozoan belonging to the trypanosomatid group that alternates between sand fly vector and mammalian hosts. They are known to cause a wide spectrum of tropical human diseases collectively known as leishmaniasis. The severity of the disease depends on the *Leishmania* species and is manifested by a range of symptoms that varies from disfiguring skin lesions to life-threatening infection of the visceral organs (1). With more than a million new cases and 20,000 to 30,000 deaths every year, 12 million people are currently affected from about 100 endemic countries imposing a significant threat to the global health care system (2, 3). Once the flagellated promastigote form of *Leishmania* enters mammalian host *via* sand fly bite, they are rapidly phagocytosed by macrophages (4). Phagocytosis occurs either directly or by engulfment of the parasite-harboring apoptotic neutrophils followed by transformation from promastigotes into nonflagellated amastigotes in the phagolysosome. Within the acidic phagolysosomes, they continue to proliferate until the cell bursts, leading to the spread of the infection (5). Inside this compartment, *Leishmania* has to withstand a variety of host-induced stress factors, including free radicals, lysosomal hydrolases, and low pH (6, 7). They are not only equipped to defend against such harsh environment but also can manipulate host gene expression to their benefit (8, 9, 10). The unavailability of a vaccine, along with increasing resistance of the existing drugs, makes it even more important to understand *Leishmania* physiology and the molecular mechanisms that allow them to thrive inside the host (11, 12).

Copper (Cu) is an essential micronutrient for biological system. Several enzymes involved in catalyzing biochemical processes utilize the ability of Cu to cycle between cuprous and cupric states. Shuttling between Cu(II) and Cu(I) states can lead to oxidative damage of cells *via* Fenton-like reaction when free Cu is available (13). Organisms have evolved mechanisms where several proteins are involved in tightly regulating the bioavailability of Cu (14). Cu homeostasis, which includes regulating its transport and intracellular distribution, is crucial as excess Cu can be detrimental. Cu-binding proteins, chaperones, and transporters keep intracellular free Cu at a very low level in the order of 10^−18^ M (15).

Various studies have shown that Cu plays a key role in host–pathogen interaction. Cu-deficient hosts are more susceptible to several pathogens, which include prokaryotes like *Salmonella typhimurium* and *Pasteurella hemolytica*, and eukaryotes like *Candida albicans* and *Trypanosoma lewisi* (16, 17, 18, 19). The bactericidal activity of macrophages and neutrophils is also impaired upon Cu deficiency (20, 21). Similarly, Cu channelization to the phagosomal compartment of macrophage during *Mycobacterium avium* and *Escherichia coli* infection indicated how hosts tend to utilize Cu to fight off intracellular pathogens (22, 23). *E. coli* mutant with a defective Cu exporting system showed significantly more susceptibility to Cu-mediated killing within macrophage (23).

The P-type Cu-ATPases are involved in exporting excess Cu from the cell and are the key players in maintaining Cu homeostasis. In bacteria, CopA and CopB are the Cu-ATPases that carry out this function (24, 25). There is a single Cu-ATPase in lower eukaryotes (nonchordates) referred to as Ccc2p in yeast *Saccharomyces cerevisiae* or ATP7 in *Drosophila melanogaster* or simply Cu-ATPase (26). With increased complexity, ATP7A and ATP7B are branched out of ATP7 in higher eukaryotes (27). In the present study, using cell-based and mouse models, we have functionally characterized the Cu-transporting ATPase (LmATP7) of *Leishmania major* and determined its role in leishmanial survivability in host macrophage. Our study establishes the physiological function of a full-length Cu-ATPase belonging to the Kinetoplastida order. In addition, our study reveals the protective role of LmATP7 in free-living promastigotes in Cu stress. We also establish that intracellular amastigotes combat host-induced Cu stress in lysosomes using LmATP7 that plays a key role in determining its pathogenicity, establishment in the host, and successful manifestation of infection.

## Results

### *Leishmania* genome encodes and expresses P-type Cu-ATPase

**Figure 1:**
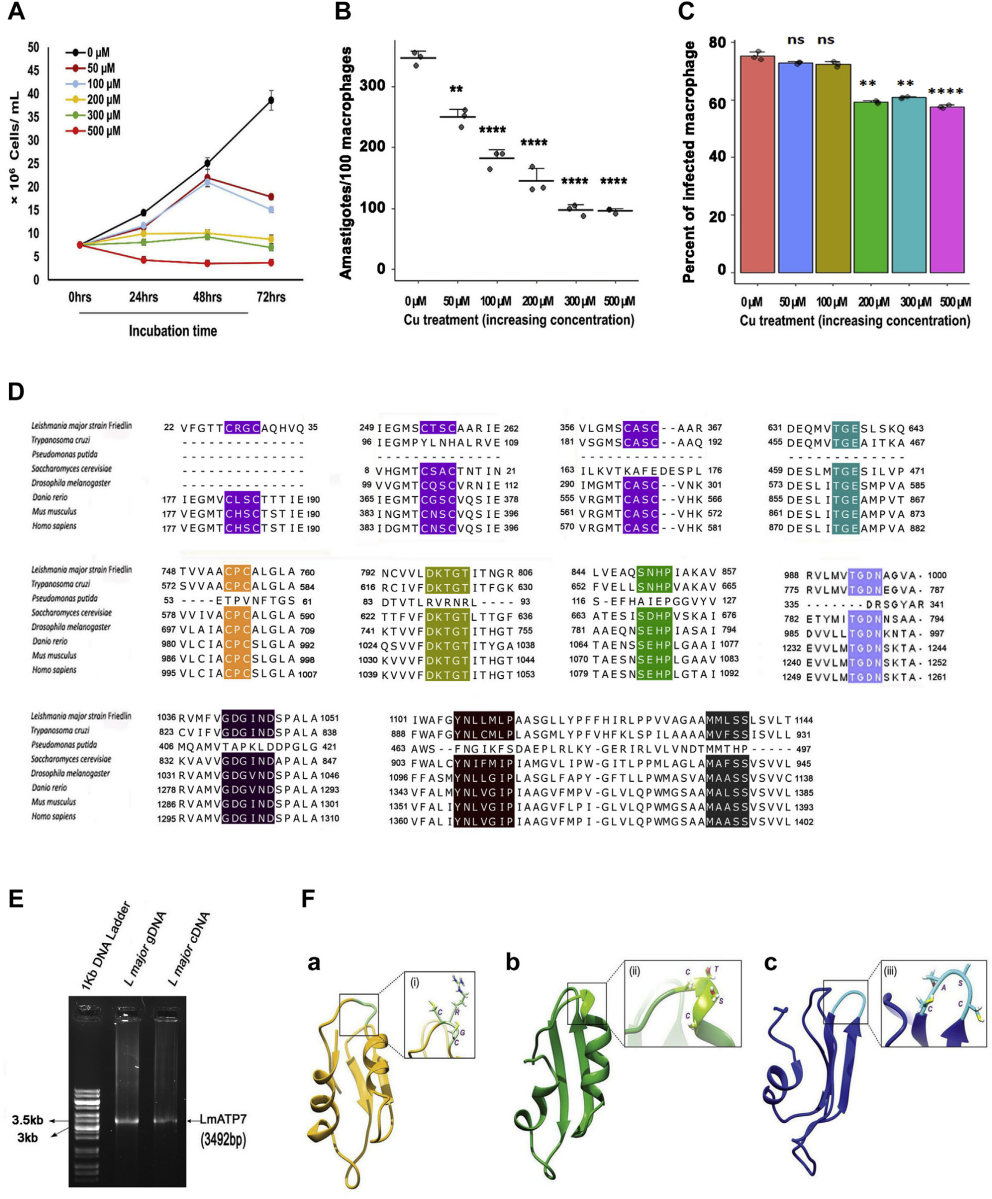
**Survivability and growth of *Leishmania major* promastigotes and amastigotes at increasing copper (Cu) concentration and identification of the putative Cu-ATPase.***A*, Wt *L. major* promastigotes were grown at 0 to 500 μM externally added Cu concentration for 24, 48, and 72 h. Initially, 7.5 × 10^6^ cells were added to the media at time point 0 h, and at the aforementioned time points, cells were counted using trypan-blue exclusion method by hemocytometer-based cell counting. Error bars represent mean ± SD of values from three independent experiments. *B*, J774A.1 macrophages were infected with Wt *L. major*. Amastigote counts inside the macrophage at 0 to 500 μM of Cu concentration at 24 h postinfection are plotted. Error bars represent mean ± SD of values from three independent experiments. ∗∗*p* ≤ 0.01, ∗∗∗∗*p* ≤ 0.0001; (Student's *t* test). *C*, the percent of infected macrophages (no. of infected macrophage cells/100 macrophages) was quantified by counting the total number of DAPI-stained nuclei of infected macrophages in a field. At least 100 macrophages were counted from triplicate experiments. Error bars represent mean ± SD of values calculated from at least three independent experiments. ∗∗*p* ≤ 0.01, ∗∗∗∗*p* ≤ 0.0001, ns (Student's *t* test). *D*, amino acid sequence alignment of the putative Leishmanial Cu-transporting P-type ATPase (accession number: XP_001685970.1) with Cu-transporting P-type ATPases from *Trypanosoma cruzi* (XP_810568.1), *Pseudomonas putida* (AAP88295.1), *Saccharomyces cerevisiae* (AJU90155.1), *Drosophila melanogaster* (NP_001259466.1), *Danio rerio* (NP_001036185.1), *Mus musculus* (AAA57445.1), and *Homo sapiens* (AAA35580.1). Conserved CXXC motifs in the N-terminal domain, actuator (A) domain TGE loop, DKTGT motif in the phosphorylation (P) domain, and TGDN, GXGXND, and SXHP motifs in the nucleotide (N) domain ATP-binding site are highlighted. Conserved transmembrane motifs CPC, YNXXXXP, and MXXSS motifs are highlighted. *E*, agarose gel electrophoresis showing amplification of *LmATP7* gene and its transcriptional expression as indicated by 3.5 kb PCR products amplified from both total genomic DNA and complementary DNA (reverse transcribed from mRNA). *F*, homology model of N-terminal Cu-binding domains of LmATP7. a, modeled structure of HM1 domain (*yellow*) with “CRGC” motif shown in *inset*. b, modeled structure of HM2 domain (*green*) with “CTSC” motif shown in *inset*. c, modeled structure of HM3 domain (*blue*) with “CASC” motif shown in *inset*. DAPI, 4′,6-diamidino-2-phenylindole; HM, heavy metal; ns, nonsignificant.

**Supplemental Figure S1:**
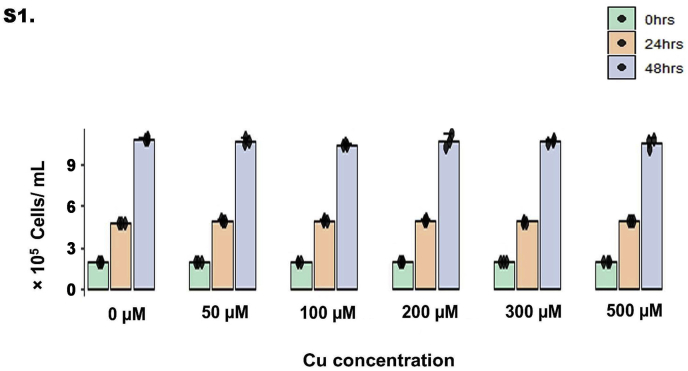
**J774A.1 macrophage cell growth in presence of external copper.** Growth of J774A.1 macrophage cells were measured in media supplemented with 0 to 500 μM of copper for 0 to 48 h. Initially, 2.0 × 10^6^ cells were added to the media (0 h) and at the above-mentioned time-points postincubation, the cells were counted using trypan-blue exclusion method by hemocytometer-based cell counting. The error bars represent mean ± SD of values from three independent experiments.

Cu has been conventionally used as a chemotherapeutic agent against parasitic infections (22, 23, 28). To explore if lethality could be induced in *Leishmania* parasites, we tested survivability of the parasite in growth media supplemented with Cu in a range between 0 and 500 μM at 24, 48, and 72 h postincubation. At 50 and 100 μM of external Cu concentration, the parasite showed a gradual decline in its growth starting from 24 h, whereas the trend was more drastic at 72 h postincubation (Fig. 1*A*). Importantly, at 200 and 300 μM of Cu concentration, there was no increase in parasite count at 24 to 72 h postincubation compared with the initial inoculum. However, at 500 μM of external Cu supplementation, the parasite number gradually decreased with time starting from 24 h postincubation indicating the toxic effect of Cu on the parasite. Interestingly, even at this high Cu concentration, the number of promastigotes remained almost same at 48 and 72 h postincubation (Fig. 1*A*). We evaluated the effect of external Cu on *Leishmania* survivability within macrophage cells. For this, we first infected J774A.1 macrophages with *L. major* promastigotes and then supplemented the growth medium with 0 to 500 μM of Cu. We checked whether Cu has any toxicity over macrophage growth but could not find any alteration when J774A.1 cells were treated with 0 to 500 μM of external Cu for 0 to 48 h (Fig. S1). However, in line with our observation on Cu-induced toxicity on free-living promastigotes, we found a similar trend in survivability of intracellular amastigotes with increasing Cu concentration. As evident from Figure 1*B*, starting from 50 μM of Cu, the parasite burden decreased significantly with about threefold reduction at 500 μM external Cu compared with untreated control 24 h postinfection (p.i.). There was a slight decline in the percentage of infected macrophages at very high external Cu concentrations (Fig. 1*C*). However, given that the internal Cu in the parasite is maintained at an extremely low level (∼3–4 ppb as measured in promastigotes using inductively coupled plasma optical emission spectrometry [ICP–OES]), the survivability trend indicated that *L. major* exhibits Cu resistance or tolerance to an appreciable extent. Internal Cu content of *L. major* is relatively low as compared with other pathogenic protozoa like *Naegleria fowleri* whose internal Cu amounts to about 84.1 ppb (29). We argue that an efficient Cu handling machinery must be present in the parasite that confers its survivability in elevated nonphysiological Cu.

We attempted to identify if *Leishmania* genome encodes for any Cu exporter that might be key for its survival in Cu stress within the macrophage lysosomes during infection or in artificial experimental condition as tested in the previous section (as shown in Fig. 1, *A*–*C*). Upon scanning the kinetoplastid informatics resources, TriTrypDB, we came across a putative Cu-transporting P-type ATPase (LmATP7) in the genome of *L. major*. Previously, Isah *et al.* (30) have used this particular ATPase in phylogenetic analysis. It is predicted to have three heavy metal (HM)-binding domains in its N-terminal region, each containing the characteristic Cu^+^-binding motif, CXXC. It has the highly conserved TGE sequence in the actuator (A) domain and DKTGT sequence in the phosphorylation (P) domain. The catalytic activity of Cu-ATPases is exhibited through phosphorylation and dephosphorylation of Asp residue in the conserved DKTGT motif. LmATP7 is also comprised of a hydrolase region containing TGDN and GXGXND conserved motifs in the ATP-binding site of the nucleotide-binding domain and the SXHP motif whose mutation in human ortholog ATP7B (His1069Gln) is the most frequent contributor to Wilson's disease in the Caucasian population (Fig. 1*D*).

The genomic DNA and RNA of *L. major* were isolated and amplified (complementary DNA [cDNA] from RNA) using primers designed to amplify the *LmATP7*. Gel electrophoresis data showed a fragment of about 3500 bp for both genomic DNA and cDNA, indicating its transcriptional expression (Fig. 1*E*), which was subsequently confirmed by Sanger sequencing. The sequencing result revealed a DNA fragment of 3492 bp, identical to the putative Cu-transporting ATPase-like protein-encoding gene (*L. major* strain Friedlin; GeneID: 5654633). Although both encoded identical amino acid sequence (1163 in length), *LmATP7* gene from our *L. major* strain 5ASKH differed in six nucleotides from the sequence of Friedlin strain. Both gene and mRNA sequences of *LmATP7* are submitted to the GenBank (accession numbers: MW261996 and MW261995, respectively).

**Supplemental Figure S2:**
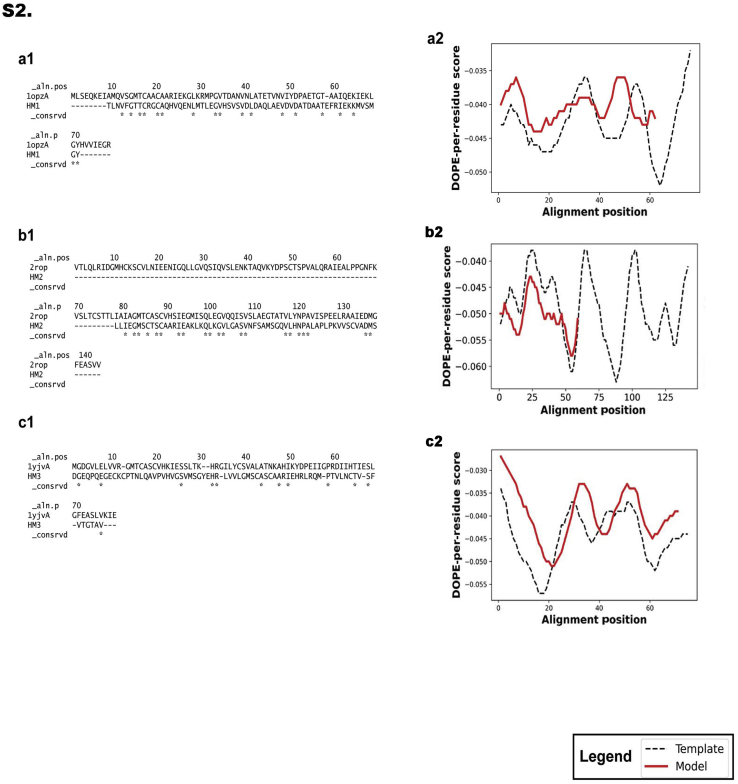
**Homology model of the Copper-binding motifs of LmATP7 protein.***A*1, sequence comparison between HM1 and 1OPZ_A chain with the conserved residues being indicated by “∗” symbol. *A*2, DOPE-per-residue score versus Alignment Position plotted for HM1 (model) and template (1OPZ.pdb). *B*1, sequence comparison between combined HM2 and 2ROP_A chain with the conserved residues being indicated by “∗” symbol. *B*2, DOPE-per-residue score versus Alignment Position plotted for HM2 (model) and template (2ROP.pdb). *C*1, sequence comparison between combined HM3 and 1YJV_A chain with the conserved residues being indicated by “∗” symbol. *C*2, DOPE-per-residue score versus Alignment Position plotted for HM3 (model) and template (1YJV.pdb).

We detected three putative HM motifs with the conserved Cys-X-X-Cys motif in LmATP7. The sequence of the three domains used for homology modeling is included in the Experimental procedures section. Based on the E value of alignment (0.65E^−04^), the template chosen for homology modeling of HM1 was *Bacillus subtilis* CopA (Protein Data Bank [PDB] ID: 1OPZ), with the percentage sequence identity between the template and the model normalized by the lengths of the alignment being 30. For HM2, based on the lowest E value of alignment (0.32E^−06^), the template that was found suitable for homology modeling was the solution structure of the metal-binding domain 4 of human ATP7B (PDB ID: 2ROP) with the percentage sequence identity between the template and the model normalized by the lengths of the alignment being 36. For HM3, based on the lowest E value of alignment (0.68E^−04^), the template that was found suitable for homology modeling was the solution structure of the metal-binding domain 6 of human ATP7A (PDB ID: 1YJV) with the percentage sequence identity between the template and the model normalized by the lengths of the alignment being 46. The modeled structures with the lowest Discrete Optimization Protein Energy score of −4818.916016, −4788.835449, −5403.311035 for HM1, HM2, and HM3, respectively, were selected for validations and simulation studies (Figs. 1*F*, S2, *A*1 and *A*2 for HM1, S2, *B*1 and *B*2 for HM2, and S2, *C*1 and *C*2 for HM3). P-type ATPases of the class IB are HM-transporting ATPases characterized by the unique presence of HM-binding sites containing Cys-X-X-Cys motifs at the cytosolic N-terminal domain. Mammalian Cu-ATPases harbors six such motifs on the amino terminus. This motif sequesters Cu and facilitates its transport across the membrane to the lumen (31). Using homology modeling based on structures of the amino-terminal HM domains of metal ATPases available in the protein database (PDB), we determined that the leishmanial ATPase has characteristic Cu^+^-binding pockets in its N terminal that share high homology with mammalian Cu-ATPases (Fig. 1*F*).

### LmATP7 primarily localizes at the plasma membrane in *Leishmania* promastigote

**Figure 2:**
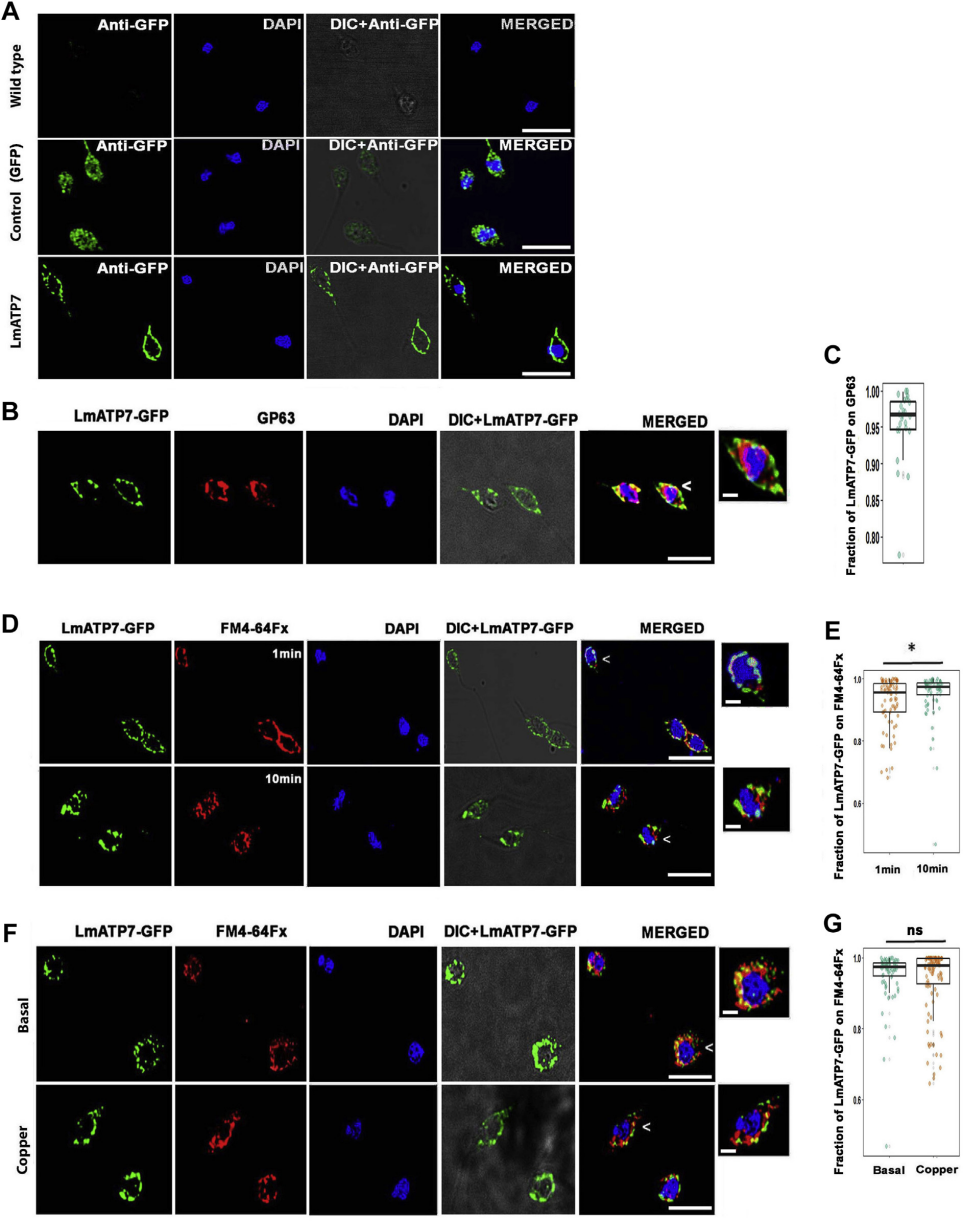
**Intracellular localization and expression of LmATP7.***A*, representative image of Wt, GFP vector control (control), and *Leishmania major* promastigotes stably expressing LmATP7 as a C-terminal GFP-tagged protein (LmATP7) immunostained with antibodies against GFP (*green*). Promastigote nuclei were stained with DAPI (*blue*). The scale bar represents 5 μm. *B*, representative image of *L. major* cells stably expressing LmATP7-GFP costained for GFP (*green*) and plasma membrane marker GP63 (*red*). The merged image shows colocalization of proteins (*yellow*). DAPI (*blue*) was used to stain the nucleus. The scale bar represents 5 μm. The magnified *inset* corresponds to the region of the merged image marked by *arrowhead* indicating the association of LmATP7-GFP and GP63. The scale bar represents 1 μm. *C*, fraction of LmATP7-GFP colocalization with GP63, demonstrated by a box plot with jitter points. The box represents the 25 to 75th percentiles, and the median in the *middle*. The whiskers show the data points within the range of 1.5× interquartile range from the first and third quartile. Sample size (n): 114. *D*, representative image of *L. major* cells stably expressing LmATP7-GFP costained for GFP (*green*) and FM4-64FX (*red*) (1 min or 10 min incubation). The merged images show colocalization of proteins (*yellow*). DAPI (*blue*) was used to stain the nucleus. The scale bar represents 5 μm. The magnified *inset* corresponds to the region of the merged image marked by *arrowheads* indicating the association of LmATP7-GFP and FM4-64FX. The scale bar represents 1 μm. *E*, fraction of LmATP7-GFP colocalization with FM4-64FX, demonstrated by a box plot with jitter points. The box represents the 25 to 75th percentiles, and the median in the *middle*. The whiskers show the data points within the range of 1.5× interquartile range from the first and third quartile. Sample size (n) for 1 min: 93 and 10 min: 102. ∗*p* ≤ 0.05 (Wilcoxon rank-sum test). *F*, representative image of *L. major* cells stably expressing LmATP7-GFP under basal and copper-treated conditions (50 μM for 2 h) costained for GFP (*green*) and FM4-64FX (*red*) (10 min). The merged images show colocalization of proteins (*yellow*). DAPI (*blue*) was used to stain the nucleus. The scale bar represents 5 μm. The magnified *inset* corresponds to the region of the merged image marked by *arrowheads* indicating the association of LmATP7-GFP and FM4-64FX. The scale bar represents 1 μm. *G*, fraction of LmATP7-GFP colocalization with FM4-64FX, demonstrated by a box plot with jitter points. The box represents the 25th to 75th percentiles, and the median in the *middle*. The whiskers show the data points within the range of 1.5× interquartile range from the first and third quartiles. Sample size (n) for basal: 117 and copper treated: 113. ns (Wilcoxon rank-sum test). DAPI, 4′,6-diamidino-2-phenylindole; ns, nonsignificant.

The human orthologs ATP7A and ATP7B localize at the *trans*-Golgi network (TGN) and traffic in vesicles to the plasma membrane upon Cu treatment (32, 33). To determine if LmATP7 localizes in the plasma membrane of *Leishmania*, we cloned and expressed *LmATP7-GFP* in promastigotes (Fig. 2*A*) and colabeled it with a *bona fide Leishmania*-specific plasma membrane marker GP63 (Fig. 2*B*). High colocalization of the two proteins was observed, with a small fraction of intracellular puncta of LmATP7-GFP that did not localize with the marker (Fig. 2, *B* and *C*). LmATP7-GFP staining by anti-GFP was specific and distinct from that of the control parasites that overexpressed only GFP (mentioned as control in Fig. 2*A*) and showed a nonspecific signal that was distributed throughout the cell. The Wt parasite did not show any GFP signal (Fig. 2*A*). To substantiate our findings, FM4-64FX was utilised to determine LmATP7-GFP localization at two time points of the dye uptake, 1 and 10 min (Fig. 2, *D* and *E*). FM4-64FX dye labels the plasma membrane at an early time point (1 min), and with time, it is endocytosed where it labels the intracellular vesicles (10 min). A high colocalization coefficient was recorded between the marker and transporter at both time points, with 10 min time point showing statistically more marker–transporter association than 1 min time point (Fig. 2*E*). This is suggestive of plasma membrane as well as endocytic vesicular localization of the protein. LmATP7 in the intracellular puncta could account for its secretory functions, including Cu delivery to Cu-requiring proteins. Upon 2 h of 50 μM Cu treatment, at the 10 min time point of FM4-64 uptake, we observed a high persistence of LmATP7 and dye colocalization similar to basal Cu condition (Fig. 2, *F* and *G*), indicating there is no appreciable change in LmATP7 localization upon Cu treatment.

### LmATP7 is a *bona fide* Cu transporter

**Figure 3:**
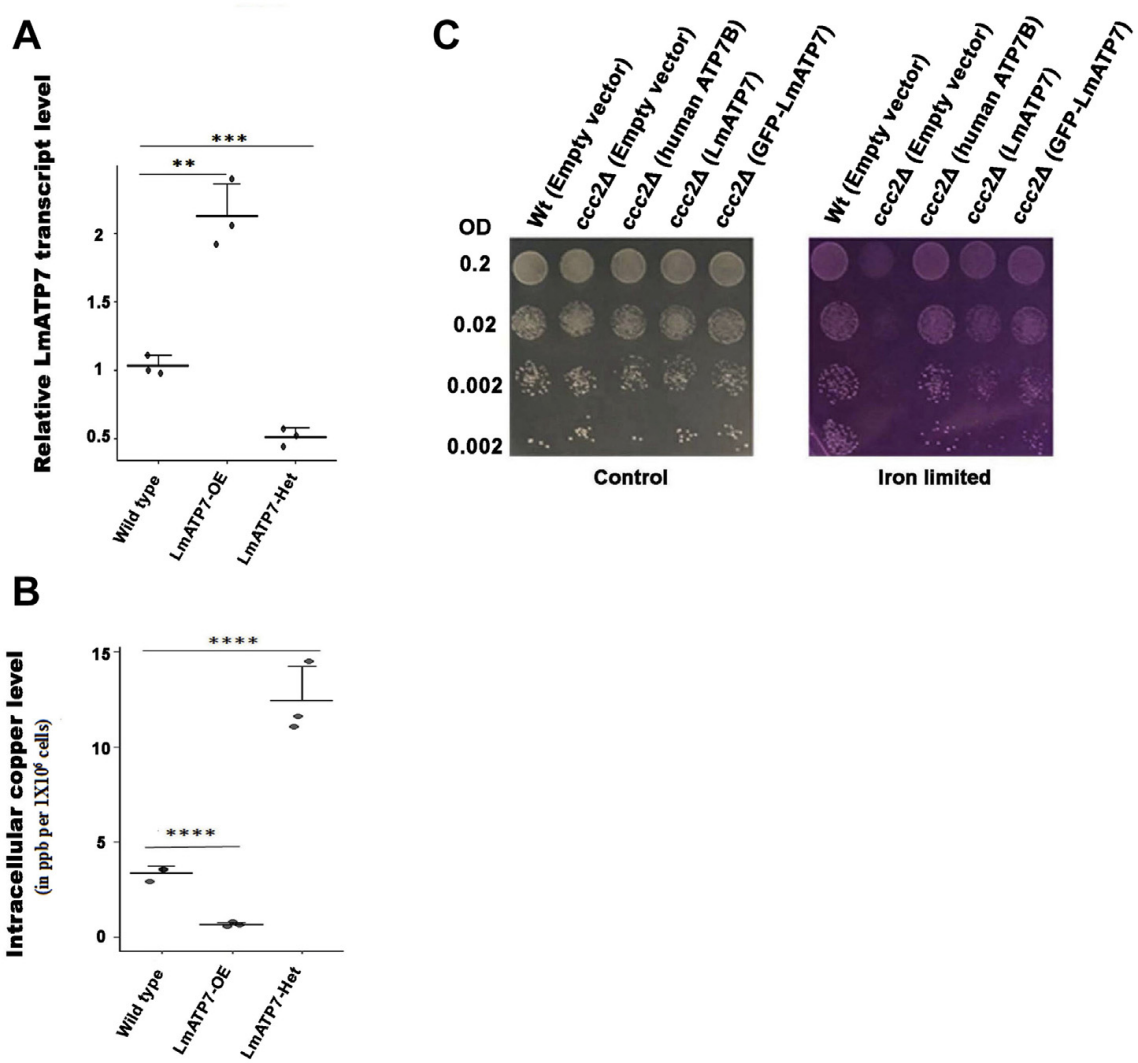
**LmATP7 transports copper.***A*, real-time PCR confirms the generation of LmATP7 overexpressed (LmATP7-OE) and heterozygous deletion (LmATP7-Het) *Leishmania major*. *LmATP7* transcript level was normalized against *rRNA45* in Wt, LmATP7-OE, and LmATP7-Het *L. major*. Error bars represent mean ± SD of values calculated from three independent experiments. ∗*p* ≤ 0.01, ∗∗∗*p* ≤ 0.001, ∗∗∗∗*p* ≤ 0.0001; (Student's *t* test). *B*, comparison of intracellular copper content (in ppb) of 1 × 10^6^ cells of Wt, LmATP7-OE, and LmATP7-Het *L. major* as measured by ICP–OES. Error bars represent mean ± SD of values from three independent experiments. ∗∗∗∗*p* ≤ 0.0001 (Student's *t* test). *C*, complementation of ccc2 mutant yeast by LmATP7. Wt and deletion (ccc2Δ) yeast strains were transformed with constructs mentioned within the parentheses. Dilution spotting was performed on SD-Ura plate (control) and 250 μM ferrozine-treated SD-Ura plate (iron limited) with yeast transformants (absorbance at 600 nm = 0.2, 0.02, 0.002, and 0.0002). Images of the plates were taken after 3 days of incubation at 30 °C. ICP, inductively coupled plasma; OES, optical emission spectrometry; SD-Ura, SD medium without uracil.

**Supplemental Figure S3:**
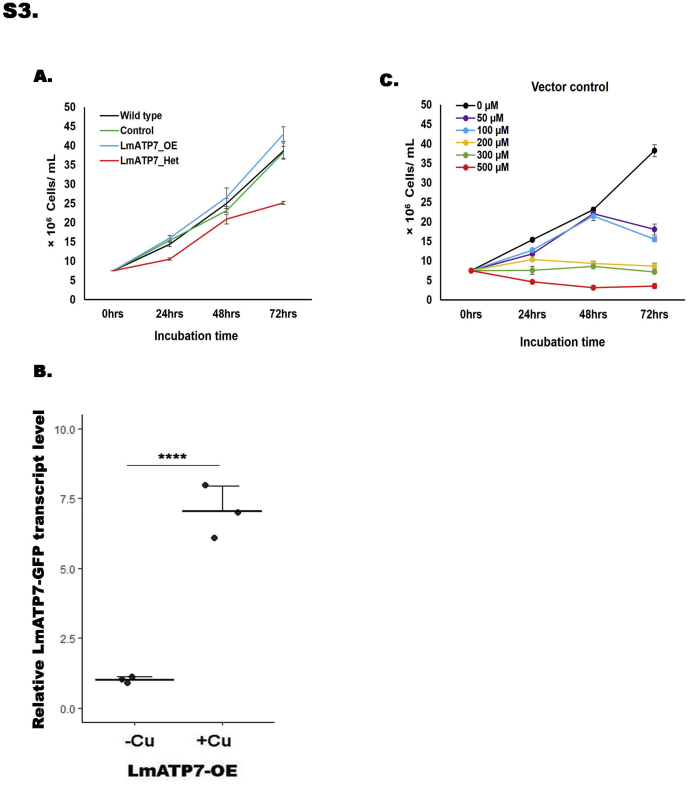
**Growth kinetics and LmATP7 transcript level in the absence or presence of external copper.***A*, growth kinetics of Wt, vector control (Control), LmATP7 overexpressing (LmATP7_OE), and LmATP7 heterozygous deletion (LmATP7_Het) *Leishmania* strains. 7.5 × 10^6^ cells were added to the media at time point 0 h and at 24 h, 48 h, and 72 h, the cells were counted using trypan-blue exclusion method by hemocytometer-based cell counting. The error bars represent mean ± SD of values from three independent experiments. *B*, RT-PCR showing LmATP7-GFP transcript level in LmATP7 overexpressed (LmATP7-OE) *Leishmania major* promastigotes grown either normally or in presence of 50 μM of external copper for 2 h. The expression of *LmATP7* was normalized against *rRNA45* gene. The error bars represent mean ± SD of values calculated from three independent experiments. ∗∗∗∗*p* ≤ 0.0001; (Student's *t* test). *C*, growth kinetics of vector control *L. major* promastigotes in presence of external copper. pXG-GFP vector control *L. major* cells were grown in 0 to 500 μM externally added copper concentration for 24 h, 48 h, and 72 h. Initially, 7.5 × 10^6^ cells were added to the media at time point 0 h and at the above mentioned time-points, the cells were counted using trypan-blue exclusion method by hemocytometer-based cell counting. The error bars represent mean ± SD of values from three independent experiments.

We hypothesized that if LmATP7 functions as a Cu transporter, then parasites overexpressing the protein should have a more efficient Cu export mechanism than the Wt and even more than the heterozygous deletion counterpart. We successfully generated a GFP-tagged *LmATP7* overexpressing (LmATP7-OE) line and a heterozygous mutant strain of LmATP7 using pXG-NEO (Figs. 3*A* and S3*A*). Our repeated attempts to develop the complete knockout of this gene in *L. major* failed, strongly indicative of the crucial role of this gene in parasite survivability. The essential nature of LmATP7 could be about not only maintaining internal Cu level but also acting as a Cu supplier to Cu-requiring and essential proteins. ICP–OES was performed to determine the intracellular Cu level of Wt, LmATP7-OE, and *LmATP7* heterozygous deletion (LmATP7-Het) *L. major* promastigotes. Higher expression of *LmATP7* was confirmed in promastigote population that stably overexpressed *GFP-LmATP7*, whereas the transcript level dropped to half in heterozygous deletion population as compared with the Wt (Fig. 3*A*). Cu level was approximately threefold less in the LmATP7-OE strain than in the Wt parasite (Fig. 3*B*). On the other hand, the LmATP7-Het strain accumulated more than threefold Cu compared with the Wt. Both results are suggestive of the role of LmATP7 in transporting Cu out of the cell.

To determine if LmATP7 is a *bona fide* Cu-ATPase, we used a functional assay that has been developed for human P-type Cu-ATPases, ATP7A/B and its mutants. This assay is based on functional complementation of the CCC2 deletion mutant (*ccc2Δ*) of the yeast *S. cerevisiae* (34). Ccc2p, the P-type Cu-ATPase transporter of *S. cerevisiae*, transports Cu to the plasma membrane oxidase Fet3p, which then works with a high-affinity iron importer Ftr1p to import iron from the medium. In the yeast deletion mutant *ccc2Δ*, Cu is not incorporated into Fet3p, leading to a deficiency in high-affinity iron uptake. In an iron-sufficient medium, the *ccc2Δ* can grow well owing to the ability of low-affinity iron uptake systems to supply iron to the cells. However, in an iron-limited medium, this mutant fails to grow as Fet3p-Ftr1p, which becomes essential under these conditions, is nonfunctional. ATP7B, in previous studies, has been shown to complement the *ccc2Δ* mutation allowing the mutant cells to grow in iron-limited medium, establishing this as a convenient functional assay for P-type Cu-ATPases.

The *S*. *cerevisiae ccc2Δ* mutant was accordingly transformed with either *LmATP7* and *GFP-LmATP7* expressed downstream of the strong TEF (Translation Elongation Factor) promoter in a single copy centromeric vector (p416TEF-*LmATP7*, p416TEF-*GFPLmATP7*) or the empty vector pTEF416 or the human *ATP7B* that was similarly expressed as a positive control (p416TEF-*ATP7B*). The transformants that appeared were evaluated for growth in iron-deficient and iron-sufficient (control) media by serial dilution assay. We observed that expression of LmATP7 and GFP-LmATP7 in *ccc2Δ* was able to complement the mutation restoring their growth in the iron-limited medium. In contrast, the cells bearing the vector control failed to grow. The p416TEF-*ATP7B* construct complemented more strongly as compared with LmATP7 and GFP-LmATP7 and may reflect either difference in expression of the two genes in yeast or possible differences in functional efficiency. Importantly, LmATP7 showed complementation, indicating that this leishmanial protein was able to functionally complement *CCC2 in vivo* (Fig. 3*C*). Hence, *LmATP7* can be functionally ascertained as the P-type of the class IB Cu-transporting ATPase in *L. major*. Since GFP-tagged LmATP7 has been used in other cell-based experiments in this study, we ensured that the GFP chimera can complement *CCC2* indicating that GFP did not interfere with the correct LmATP7 localization on which its functioning depends.

### LmATP7 imparts Cu tolerance to *L. major* promastigotes

**Figure 4:**
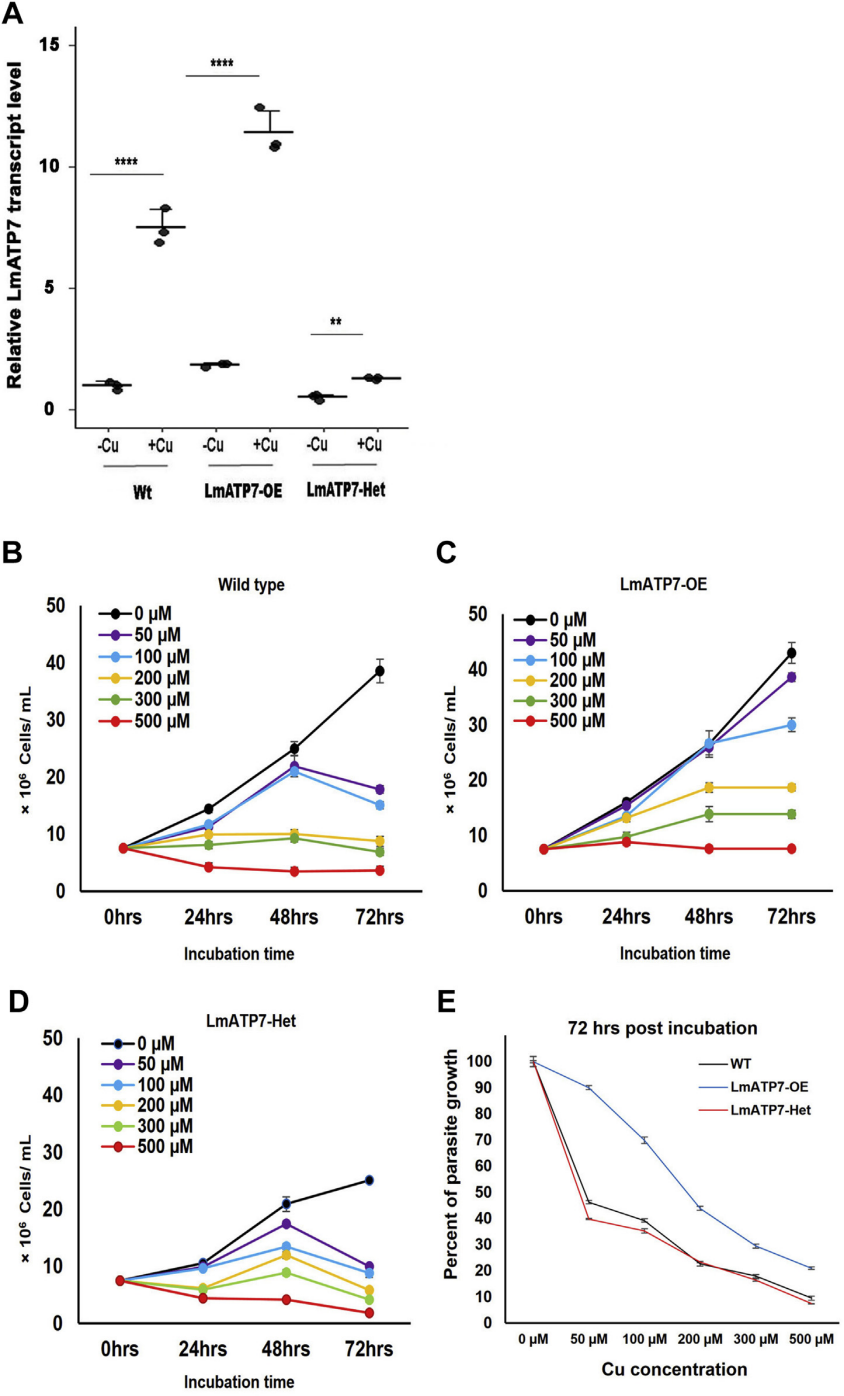
**LmATP7 imparts copper tolerance to *Leishmania major* promastigotes.***A*, RT–PCR showing *LmATP7* transcript level in Wt, LmATP7 overexpressing (LmATP7-OE), and LmATP7 heterozygous deletion (LmATP7-Het) strains of *L. major* grown either normally or in the presence of 50 μM of external copper for 2 h. Expression of *LmATP7* was normalized against *rRNA45* gene. Error bars represent mean ± SD of values calculated from three independent experiments. ∗∗*p* ≤ 0.01, ∗∗∗∗*p* ≤ 0.0001 (Student's *t* test). Wt (*B*), LmATP7-OE (*C*), and LmATP7-Het. *D*, *L. major* promastigotes were grown at 0 to 500 μM externally added copper for 24, 48, and 72 h. Initially, 7.5 × 10^6^ cells were added to the media at time point 0 h, and at the aforementioned time points, cells were counted using trypan-blue exclusion method by hemocytometer-based cell counting. Error bars represent mean ± SD of values from three independent experiments. *E*, line graph showing the comparison between growth of Wt, LmATP7-OE, and LmATP7-Het promastigotes in the presence of the indicated concentrations of external copper (0–500 μM) at 72 h postincubation. Error bars represent mean ± SD of values from three independent experiments.

Since we have ectopically expressed and established LmATP7 as a Cu-ATPase capable of Cu uptake in yeast, we now investigated if the protein can regulate Cu homeostasis in *Leishmania*. Cu treatment has been shown to upregulate ATP7A in mammalian cells (35). To determine if Cu has a role in LmATP7 regulation in *Leishmania*, we challenged Wt, LmATP7-OE, and LmATP7-Het *L. major* promastigotes with 50 μM Cu for 2 h or not and determined the transcript levels. Interestingly, in all cases, we observed an increase in the transcript level while the cells were treated with Cu, indicating the possible role of LmATP7 in maintaining *Leishmania* Cu homeostasis (Figs. 4*A* and S3*B*). To verify if this gene imparts any resistance toward Cu-mediated toxicity on the parasites, we grew Wt, vector control (GFP-only), LmATP7-OE, or LmATP7-Het promastigotes in the presence of 0 to 500 μM of external Cu and systematically observed the parasite growth kinetics at 0 to 72 h postincubation. As evident from Figure 4, *B*–*D*, both Wt and vector control (Fig. S3*C*) *L. major* strains showed reduced growth with increasing Cu in the medium starting from 24 h postincubation, indicating cytotoxic effect of excess Cu. In fact, at 500 μM concentration of external Cu, Wt and vector control promastigote counts were lower than the initial seeded number at 24 h postincubation, indicating cell death. Interestingly, LmATP7-OE promastigote count was significantly higher than the controls at such heightened Cu level throughout the time points, which could even tolerate 500 μM of external Cu stress. Although its growth decreased gradually with increasing Cu conditions, it consistently maintained a higher growth rate than the controls. This indicates an enhanced Cu export activity in LmATP7-OE promastigotes (also evident from Fig. 3*B*), allowing a relatively better internal Cu regulation and reduced cytotoxic effects. Importantly, LmATP7-Het cells struggled to grow at a comparable rate with Wt cells even in the absence of externally added Cu. Also, starting from 50 μM of Cu treatment, the cell count decreased gradually with increasing Cu concentration as well as with time. We have also shown the parasite growth between Wt, LmATP7-OE, and LmATP7-Het cells at 72 h postincubation in the presence of 0 to 500 μM of Cu concentration and illustrated our findings in Figure 4*E*. Both the controls were also able to survive and maintain their growth to some extent under excess external Cu, probably owing to their endogenously expressed LmATP7. At extremely high Cu, endogenous LmATP7 seemed insufficient to maintain internal Cu homeostasis, leading to growth retardation and cell death. The results provided the first evidence of the role of the newly characterized leishmanial Cu-ATPase, LmATP7, in Cu tolerance.

### Macrophages channelize Cu to intracellular compartments harboring *Leishmania* amastigotes

**Figure 5:**
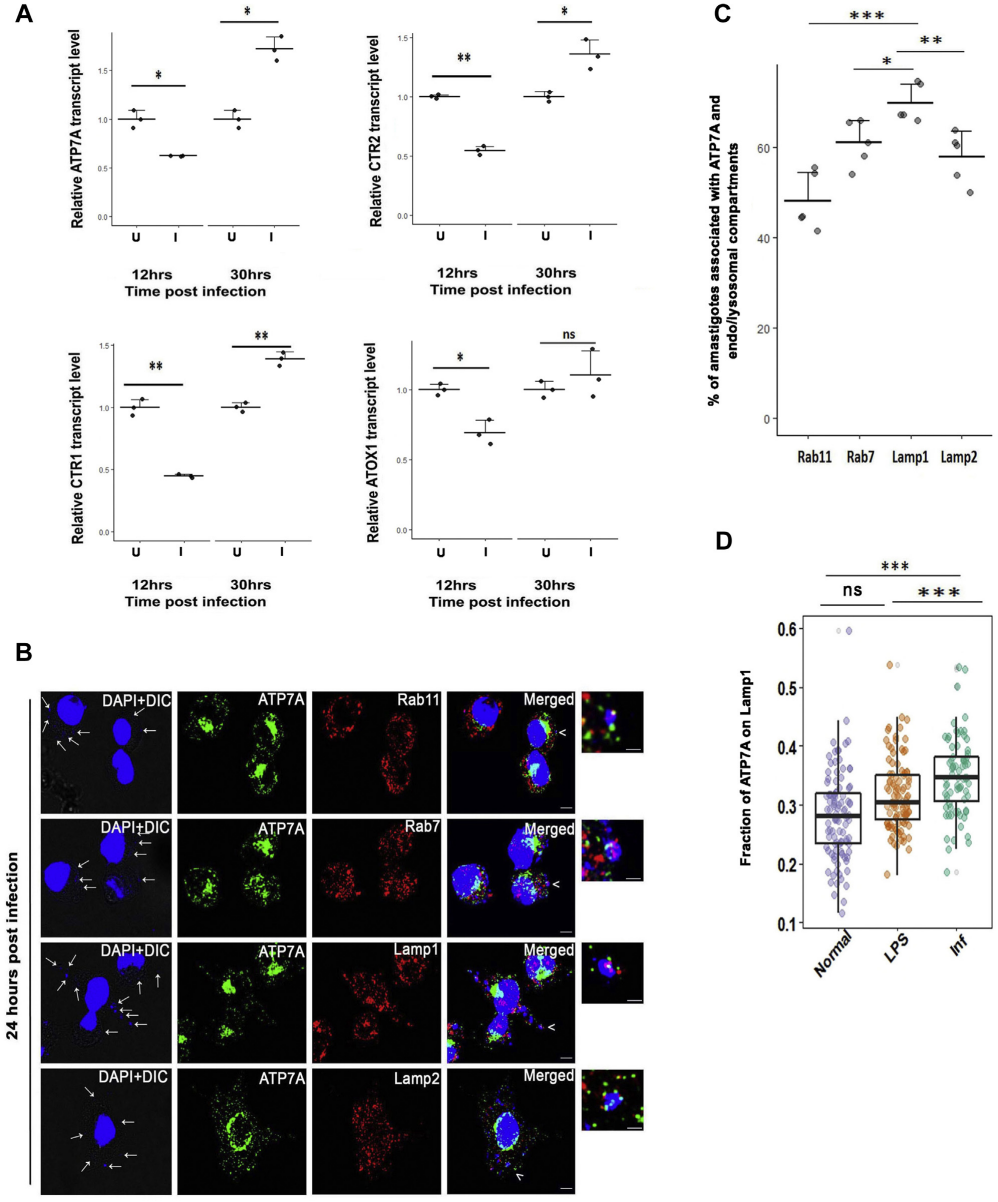
***Leishmania* infection elicits a host response that is mediated through its copper homeostasis genes.***A*, J774A.1 macrophage cells were either infected (I) or not (U) with *Leishmania major* promastigotes for 12 and 30 h. RT–PCR shows transcript levels of macrophage copper homeostasis genes *ATP7A*, *CTR1*, *CTR2*, and *ATOX1* normalized against *β-actin* mRNA levels. Error bars represent mean ± SD of values calculated from three independent experiments. ∗*p* ≤ 0.05, ∗∗*p* ≤ 0.01, ns (Student's *t* test). *B*, representative images of J774A.1 macrophages infected with *L. major* promastigotes and stained with anti-ATP7A (*green*) and anti-Rab11 (*red*) or anti-Rab7 (*red*) or anti-Lamp1 (*red*) or anti-Lamp2 (*red*) at 24 h postinfection. The merged images represent colocalization of ATP7A with *Leishmania* amastigote nuclei (*blue*) and endosomal markers (Rab11, Rab7, Lamp1, and Lamp2) (*red*). Both macrophage and *Leishmania* nuclei were stained with DAPI (*blue*). *White arrows* indicate intracellular parasites in infected cells (smaller nuclei). The scale bar represents 5 μm. The magnified *inset* corresponds to the region of the merged image marked by *arrowheads* indicating the association of ATP7A and endosomal markers with *Leishmania*-positive endosomes. The scale bar represents 1 μm. *C*, percentage of *L. major* amastigote nuclei associated with both ATP7A and the indicated endosomal markers (Rab11, Rab7, Lamp1, and Lamp2) of J774A.1 macrophages. The number of *Leishmania* nuclei counted to obtain the data for each condition are 197, 189, 274, 243 (for Rab11, Rab7, Lamp1, Lamp2, respectively). Error bars represent mean ± SD of values calculated from three independent experiments. ∗*p* ≤ 0.05, ∗∗*p* ≤ 0.01, ∗∗∗*p* ≤ 0.001, ns (Wilcoxon rank-sum test). *D*, fraction of ATP7A colocalization with Lamp1 from uninfected (normal), LPS treated (LPS), and *L. major*-infected J774A.1 macrophages (infected) demonstrated by a box plot with jitter points. The box represents the 25th to 75th percentiles, and the median in the *middle*. The whiskers show the data points within the range of 1.5× interquartile range from the first and third quartiles. Sample size (n) for normal: 83, LPS treated: 79, and infected: 75. ∗∗∗*p* ≤ 0.001, ns; (Wilcoxon rank-sum test). ATOX1, antioxidant protein 1; CTR1, Cu transporter 1; CTR2, Cu transporter 2; DAPI, 4′,6-diamidino-2-phenylindole; Lamp, lysosome-associated membrane protein; LPS, lipopolysaccharide; ns, nonsignificant.

**Supplemental Figure S4:**
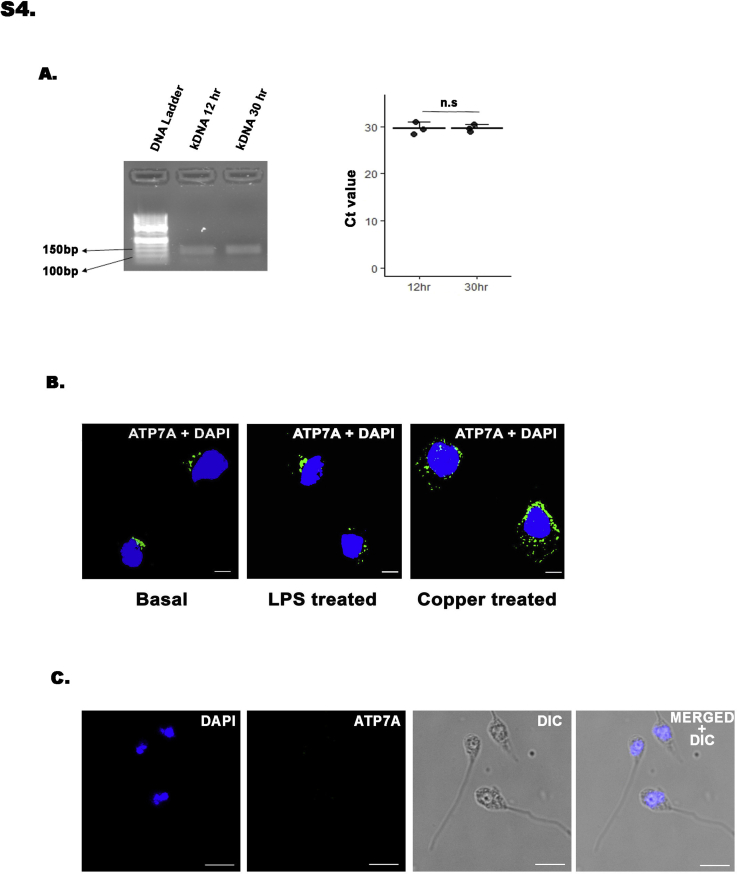
**Time-dependent infection status in macrophages and specificity of ATP7A antibody are checked.***A*, agarose gel electrophoresis showing PCR products amplified from total cDNA isolated from *Leishmania major* infected J774A.1 macrophage at 12 h and 30 h postinfection time-points with *Leishmania* kDNA specific RT-qPCR primers. Graphical representation of Ct values of kDNA transcript at 12 h and 30 h postinfection time-point. The error bars represent mean ± SD of values calculated from three independent experiments. n.s: non-significant; (Student's *t* test). *B*, representative images of Control (Basal), LPS treated (2 h), and copper treated (50 μM; 2 h) macrophages immunostained with anti-ATP7A (*green*). Nuclei were stained with DAPI (*blue*). The scale bar represents 5 μm. *C*, representative images of *L. major* promastigotes immunostained with anti-ATP7A (*green*). Nuclei were stained with DAPI (*blue*). The scale bar represents 5 μm.

To further delineate the role of LmATP7 in a physiological condition, we explored host–*Leishmania* interaction with respect to Cu stress subjected by the host upon parasite and the parasite's reciprocating response thereon to evade the stress. At the outset, we examined alterations of macrophage (host) transcript levels (if any) of the key genes (*ATP7A*, *CTR1*, *CTR2*, and antioxidant protein 1 [*ATOX1*]) of the Cu homeostatic pathway during *Leishmania* infection. ATP7A is a P-type ATPase that provides Cu to Cu-dependent enzymes. When Cu is in excess, ATP7A, a Golgi-resident protein, acts as an exporter and traffics out of Golgi in vesicles to remove the Cu from the cell (36). Cu transporter 1 (CTR1) and Cu transporter 2 (CTR2) are Cu importers located in the plasma membrane and endolysosomal membranes, respectively, whereas Atox1 is a cytosolic Cu chaperone that carries Cu from CTR1 to ATP7A (37, 38). J774A.1 macrophages were infected with *L. major* promastigotes, and infection was confirmed and measured by counting the nuclei of amastigotes in the macrophages. Transcripts of Cu-ATPase *ATP7A*, Cu transporters *CTR1* and *CTR2*, and Cu chaperone *ATOX1* were measured 12 and 30 h p.i. Interestingly, all four genes were downregulated at the shorter p.i. time point; however, at 30 h p.i., *ATP7A* exhibited ∼1.8× upregulation. Other transcripts also exhibited modest upregulation (Fig. 5*A*). There was no significant alteration in the infection pattern at 12 and 30 h p.i. as evident from *Leishmania*-specific kinetoplast DNA transcript level (Fig. S4*A*) (39). The results suggested that host Cu uptake and utilization is downregulated during the early stages of infection. However, the host exerts its Cu-mediated response at a later stage with upregulation of *ATP7A*, *CTR1*, and *CTR2*.

To export excess Cu, ATP7A traffics to vesicle out of TGN when cells are treated with high Cu (33, 40, 41). Macrophage ATP7A is capable of trafficking upon Cu treatment (Fig. S4*B*). Hence, as a function of Cu transport, we probed intracellular localization and TGN exit of ATP7A at 24 h post-*Leishmania* infection. The time point fits between the heightened parasite response and heightened host response. Interestingly, ATP7A traffics out of Golgi in vesicles in *L. major*-infected macrophages (Fig. 5*B*). ATP7A in uninfected macrophages remained clustered at the Golgi (Fig. S4*B*). There is no crossreactivity with anti-ATP7A antibody on Leishmanial protein, LmATP7 (Fig. S4*C*). Since *Leishmania*, postinternalization, localizes in the macrophages throughout the endosomal pathway, we determined the nature of the ATP7A-positive compartment that harbors this amastigote parasite. To scan the entire endocytic pathway, we measured the percentage of amastigote nuclei associated with both ATP7A and the endosomal/endolysosomal markers, Rab11 (recycling endosome), Rab7 (late endosome), and lysosome-associated membrane protein 1/2 (Lamp1/2) (endolysosome). We noticed that colocalized ATP7A-amastigote nuclei distribute throughout the endosomal pathway (Fig. 5, *B* and *C*); with the highest localization at the Lamp1-positive compartments (Fig. 5, *C* and *D*).

### LmATP7 is important for the intracellular survival of *L. major* in macrophages

**Figure 6:**
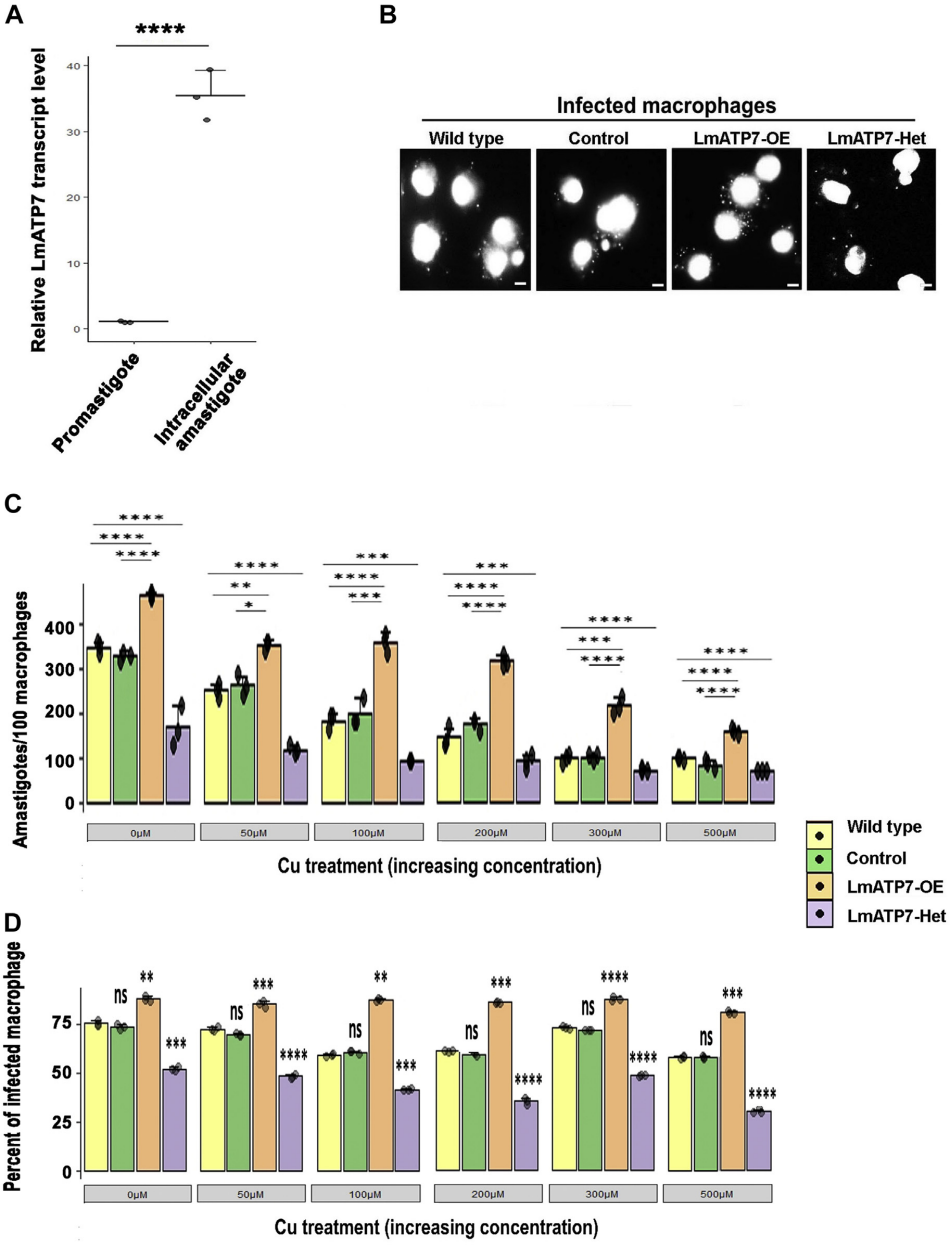
**LmATP7 is key for amastigote survivability in macrophages in basal and elevated copper.***A*, RT–PCR showing LmATP7 transcript level in Wt *Leishmania major* promastigotes and intracellular amastigotes. Expression of *LmATP7* was normalized against *rRNA45* gene. Error bars represent mean ± SD of values calculated from three independent experiments. ∗∗∗∗*p* ≤ 0.0001 (Student's *t* test). *B*, representative DAPI-stained epifluorescence microscopy images of three independent experiments showing J774A.1 macrophages infected with Wt, vector control (control), LmATP7 overexpressed (LmATP7-OE), or LmATP7 heterozygous deletion (LmATP7-Het) *L. major* strains post 24 h of infection. Larger nuclei represent macrophage cells surrounded by smaller amastigote nuclei of the parasite. Images were taken under Olympus IX-81 epifluorescence microscope at 60× magnification. The scale bar represents 5 μm. *C*, graphical representation of amastigotes/100 macrophage count at 24 h postinfection. J774A.1 macrophages were infected with Wt, vector control (control), LmATP7 overexpressed (LmATP7-OE), or LmATP7 heterozygous deletion (LmATP7-Het) strains in the presence of indicated concentrations of copper (0–500 μM). At least 100 macrophages were counted from triplicate experiments. Error bars represent mean ± SD of values from three independent experiments. ∗*p* ≤ 0.05, ∗∗*p* ≤ 0.01, ∗∗∗*p* ≤ 0.001, and ∗∗∗∗*p* ≤ 0.0001 (Student's *t* test). *D*, the percent of infected macrophages (number of infected macrophage cells/100 macrophages) was quantified by counting the total number of DAPI-stained nuclei of infected macrophages in a field. At least 100 macrophages were counted from triplicate experiments. Error bars represent mean ± SD of values calculated from at least three independent experiments. ∗∗*p* ≤ 0.01, ∗∗∗*p* ≤ 0.001, ∗∗∗∗*p* ≤ 0.0001, ns (Student's *t* test). DAPI, 4′,6-diamidino-2-phenylindole; ns, nonsignificant.

It is now obvious that as a host response, the macrophage deploys the Cu transport machinery to exert stress on the intracellular parasites. We argue that *Leishmania* uses the LmATP7 to efficiently bypass Cu stress brought upon by the host macrophage as the parasite establishes in the macrophage and morphs from the promastigote form to amastigote during their intracellular life cycle. We compared *LmATP7* expression levels between the free-living promastigote and the intramacrophagic amastigotes. Interestingly, we recorded a tremendous elevation of *LmATP7* transcript (∼35 times) in the amastigotes over the promastigote form (Fig. 6*A*). This suggests a critical role of the Cu transporter toward the establishment of *Leishmania* infection in the host macrophage.

**Supplemental Figure S5:**
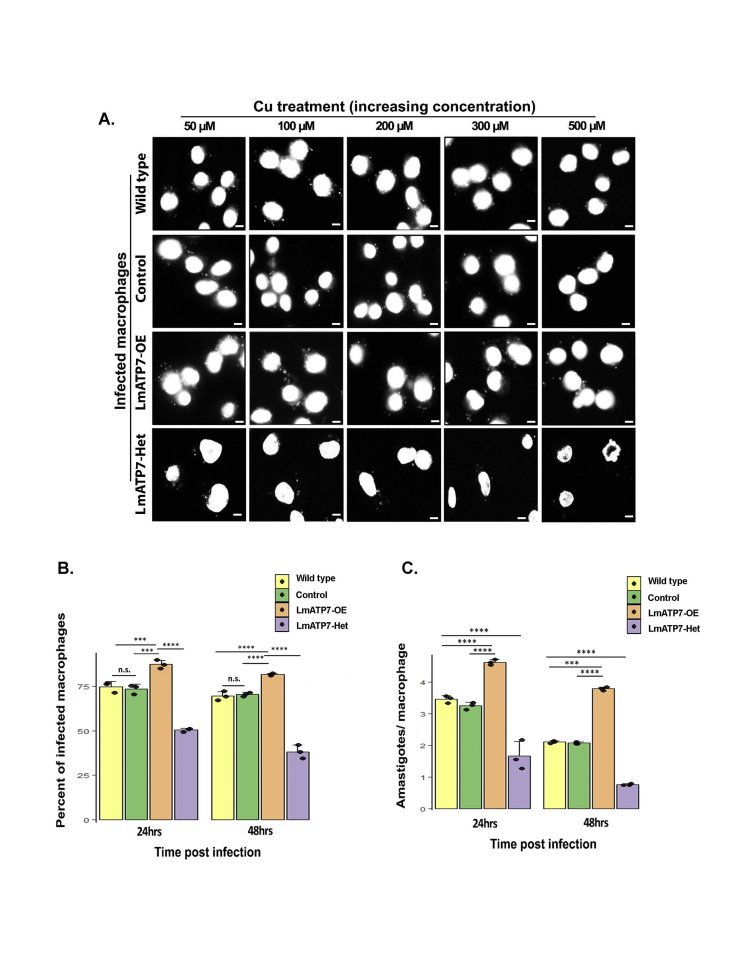
**LmATP7 gene is important for the survival and infectivity of *Leishmania major*.***A*, representative DAPI-stained epifluorescence microscopy images of three independent experiments showing J774A.1 macrophage infected with Wt, vector control (Control), LmATP7 overexpressed (LmATP7-OE), or LmATP7 heterozygous deletion (LmATP7-Het) *L. major* strains in presence of external copper (50–500 μM) at 24 h postinfection. In the images, larger nuclei represent macrophage cells which are surrounded by smaller nuclei of *Leishmania* amastigotes. Images were taken under Olympus IX-81 epifluorescence microscope at 60× magnification. The scale bar represents 5 μm. *B*, J774A.1 macrophage cells were infected with Wt, Control, LmATP7-OE, or with LmATP7-Het *L. major* promastigotes for either 24 or 48 h in the absence of externally added Cu. The cells were stained with DAPI and visualized under Olympus IX-81 epifluorescence microscope. The percent of infected macrophages (no. of infected macrophage cells/100 macrophages) was quantified by counting the total number of DAPI-stained nuclei of infected macrophages in a field. At least 100 macrophages were counted from triplicate experiments. The error bars represent mean± SD of values calculated from at least three independent experiments. ∗∗∗*p* ≤ 0.001, ∗∗∗∗*p* ≤ 0.0001, n.s: non-significant; (Student's *t* test). *C*, graphical representation of amastigotes/ macrophage count at 24 h and 48 h postinfection. J774A.1 macrophages were infected with Wt (*black bar*), Control (*yellow bar*), LmATP7-OE (*blue bar*), or LmATP7-Het (*red bar*) strains in the absence of externally added Cu. At least 100 macrophages were counted from triplicate experiments. The error bars represent mean ± SD of values from three independent experiments. ∗*p* ≤ 0.05, ∗∗*p* ≤ 0.01, ∗∗∗*p* ≤ 0.001, ∗∗∗∗*p* ≤ 0.0001; (Student's *t* test).

Subsequently, we determined the role of this Cu-ATPase in intramacrophagic survivability (p.i.) of the parasites. Stable lines of *L. major* expressing LmATP7-GFP (LmATP7-OE) or heterozygous deletion mutant LmATP7-Het were used to infect J774A.1 macrophages under normal and increased Cu conditions. Empty vector (pXG-GFP+) expressing and Wt promastigotes were used as two controls. Corroborating with our hypothesis, we found that LmATP7-OE parasites were significantly higher in abundance as compared with both the controls at 24 h p.i. (Fig. 6*B*, quantified in Fig. 6*C*, 0 μM Cu). This increase was consistent both at normal and Cu-treated conditions suggesting when overexpressed, LmATP7 by exporting Cu from *Leishmania* provided increased survivability to the amastigotes (Figs. 6*C* and S5*A*). Previous studies showed that bactericidal activity is promoted by intracellular bioavailable Cu in macrophages, which was further facilitated by external Cu treatment (23). We made a similar observation where increase in external Cu concentration led to reduced intracellular amastigote load for all the leishmanial strains. As illustrated in Figure 5*B*, under physiological Cu condition, activated macrophages increase Cu import and partially channel Cu *via* ATP7A toward the endolysosomal compartments. LmATP7 being overexpressed was probably more efficient in removing excess Cu from the *Leishmania* cells than the endogenously expressed one resulting in higher amastigote per macrophage cell (Fig. S5*B*). On the other hand, LmATP7-Het failed to establish a successful infection when externally treated with Cu. As compared with Wt control, even in the absence of externally added Cu, the parasite burden is more than twofolds low in case of LmATP7-Het. The trend remained unaltered with increasing concentration of Cu treatment (Figs. 6, *B* and *C* and S5*A*).

We also wanted to check whether this gene plays any role in parasite infectivity as well as intracellular replication of amastigotes. As shown in Fig. S5*B*, there was ∼20% increase in the percent of infected macrophages when macrophages were infected with LmATP7-OE at both 24 and 48 h p.i. compared with its vector control and Wt counterpart. LmATP7-Het parasites showed a significant ∼25% decrease in the percent of infected macrophages at 24 h p.i. compared with Wt *L. major*. In fact, at 48 h p.i., there was ∼30% decrease (Fig. S5*B*). We found a similar trend in the percentage of infected macrophages for all four *L. major* strains when treated with increasing concentration of external Cu (Fig. 6*D*).

Similar to this observation when we extended our infection study with all those four strains up to 48 h in the absence of externally added Cu, we have seen about twofold increase in parasite burden with LmATP7-OE compared with the controls. Interestingly, at that time point, while the Wt *L. major* could maintain its population successfully, LmATP7-Het cells showed about threefold decrease with the parasite burden of ∼0.7, that is, less than one amastigote/macrophage cell (Fig. S5*C*). Overall, the experimental data revealed the importance of LmATP7 in neutralizing toxic levels of Cu from the host on *Leishmania* amastigotes. Also, collectively our study establishes that LmATP7 is crucial for both the survivability as well as infectivity of *L. major* parasite.

### LmATP7 promotes *L. major* proliferation and infectivity *in vivo* in a mouse model

**Figure 7:**
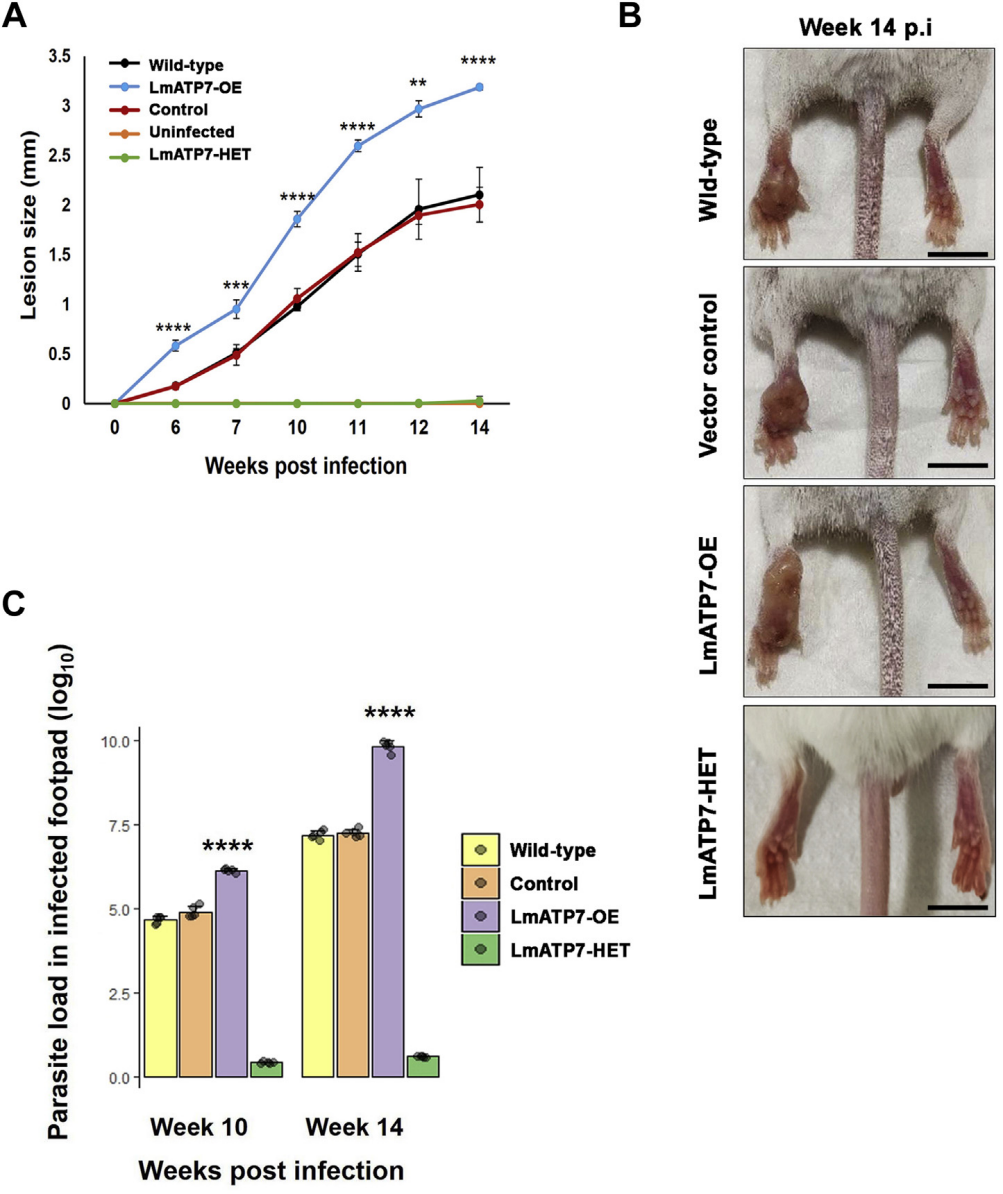
***Leishmania major* overexpressing LmATP7 exhibits heightened infection and increases survivability in the mouse model of cutaneous Leishmaniasis.***A*, BALB/c mice were inoculated into the left hind footpad with 1 × 10^6^ stationary phase promastigotes suspended into PBS of Wt, vector control (control), LmATP7 overexpressed (LmATP7-OE), and LmATP7 heterozygous deletion (LmATP7-HET) parasites or with only PBS as in uninfected control. Lesion development was determined by weekly measuring the swelling with a caliper. The data correspond to the mean ± SD of values obtained from five individual mice in each group. *B*, representative images showing the extent of lesion formation in each of the infected mouse groups at week 14 postinfection. The scale bar represents 1 cm. *C*, parasite load in the infected footpad was determined at week 10 and 14 postinfection by limiting dilution assay, which is represented as geometric means. Finally, the parasite load in the LmATP7-OE–infected mice footpad was compared with the parasite burden in Wt *L. major*–infected mice footpad. ∗∗∗∗*p* ≤ 0.0001 (Student's *t* test).

Our findings establish that *LmATP7* mediates infection and survivability of *Leishmania* in J774A.1 macrophages by evading host-induced Cu stress. We verified whether this gene is responsible for a similar outcome under *in vivo* infection condition. We infected BALB/c mice with promastigotes of Wt, vector control, LmATP7-OE, and LmATP7-Het *L. major* strains by subcutaneous injection to the left hind footpad as per the previous reports and monitored the onset and progression of lesion development in these four groups of animals up to week 14 p.i. (42). As shown in Figure 7*A*, the Wt and vector control *L. major*-infected mice started to develop visible lesion at week 6 p.i. Importantly, the vector control strain showed a similar pathology like Wt *Leishmania*, as evident from the trend of lesion development in infected mice footpad over time. In contrast to these, LmATP7-OE strain–infected mice footpad developed a heightened lesion as early as week 6 p.i., which continued to increase significantly until the end of this experiment. LmATP7-Het strain–infected mice footpad showed no noticeable lesion till the 14th week. The representative image of LmATP7-OE-infected mice footpad clearly shows that the size of lesion is significantly higher than either Wt or vector control–infected mice at week 14 p.i. (Fig. 7*B*). This comparative analysis of lesion development within infected mice footpads certainly provides a strong indication that LmATP7 plays a crucial role in imparting disease pathology by *L. major* parasites. To determine whether this increased lesion development by LmATP7-OE strain infection is indeed linked to increased proliferation of the parasite *in vivo*, we next quantified parasite burden in these four groups of infected mice footpad at week 10 p.i. (where we observed a significant increase in lesion size) and at week 14 p.i. (when there was maximum swelling). As shown in Figure 7*C*, there was no significant difference between parasite titer in mice infected with Wt and control strains. On the other hand, the LmATP7-OE strain titer in infected footpad at week 10 and week 14 p.i. was ∼2 and ∼3-logs higher, respectively, than the Wt *L. major*-infected mice. Interestingly, the LmATP7-Het strain–infected mice footpad with no visible lesion, both at week 10 and week 14 p.i., had a very low parasite titer as compared with the Wt *L. major*-infected mice.

Taken together, these findings conclusively establish that the *LmATP7* that we characterized as a Cu-ATPase is crucial in parasite infectivity, its intramacrophagic survivability, as well as replication *in vivo*.

## Discussion

Host–parasite interaction is a rapidly evolving field. Host–parasite interaction has served as an apt example of “red queen hypothesis of winner less antagonistic coevolution” (43). Host and the parasite coevolve where parasites adapt to neutralize any response employed by the host to evade parasite invasion. Macrophage cells, being the sentinel of our immune system, play pivotal role in eliminating different pathogenic infections (44). However, *Leishmania* parasites specifically infect and survive within these cells. Macrophages have been shown to express the key Cu-homeostatic genes. It has been demonstrated that in the case of pathogenic yeast, *Candida* sp. infection, macrophage deploys the Cu-ATPase, ATP7A, to exert Cu-mediated toxicity over the pathogen (45). However, there is no such report that addresses if macrophage being the host to *Leishmania* tries to combat the infection using similar mechanism and, if so, then how the parasite escapes such host-induced stress. In this context, we have characterized the Cu-ATPase, LmATP7, for the first time in the kinetoplastid parasite genus, *Leishmania*. Furthermore, using the well-established macrophage–*Leishmania* infection model system, we found that there is a reciprocal interaction between the Cu-ATPases of the host and the parasite that determines the fate of parasite survivability and well-being of the antagonistic pair.

We observed that upon increasing concentration of Cu treatment, Wt *L. major* promastigotes could overcome the toxic effect of this metal ion. This observation is particularly interesting since the other known bacterial pathogens are reported to be susceptible to Cu concentration as low as 20 μM (23). Although at 50 μM and higher external Cu treatment, there was a drop in parasite count, *Leishmania* could maintain its population appreciably at a very high Cu concentration of 500 μM. We noticed a similar survival trend even in the intracellular amastigotes in these Cu conditions. These two exciting observations collectively suggest that there must be an efficient Cu homeostasis mechanism within *Leishmania*, which is expressed both in its extracellular promastigote form as well as in its infectious amastigote stage.

Importance of Cu in kinetoplastid parasites is unknown. Though the cytochrome C oxidase complex (known to bind Cu) has been identified in *Leishmania* and its component Ldp27 has been shown to be crucial in amastigote survival, no information is available about the role of Cu in the secretory pathway (46). Meade *et al.* (47) in a comprehensive review enlisted a spectrum of identified P-type ATPases in pathogenic trypanosomes and *Leishmania*. The presence of a Cu/Zn-type superoxide dismutase in *Leishmania* glycosomes may indicate the role of a Cu P-type ATPase in incorporating Cu to TGN and intracellular organelles. Although the role of iron has been extensively studied in kinetoplastid parasites, there have been limited efforts to understand the role of Cu in their survival or pathogenicity in this order as well as protozoal parasite as a whole. Pathogenic protozoa like *N. fowleri* and *Plasmodium falciparum* use their Cu-ATPase to detoxify Cu (29, 48). *C. albicans* also employ this transporter to maintain Cu homeostasis (49).

Well-characterized mammalian Cu-ATPase, ATP7A, carries out two primary functions. First, it transports Cu to enzymes that require the metal as a cofactor for maturation, for example, dopamine-β-hydroxylase and tyrosinase. Second, upon Cu excess in the cell, ATP7A traffics in vesicles to export out the excess Cu (33). In a polarized epithelial cell, as in intestinal enterocytes, ATP7A transports Cu through the basolateral surface (blood side) that is picked up in the serum by Cu-sequestering proteins, for example, albumin. However, in unpolarized cells, for example, macrophages and fibroblasts, the Cu transporter vesicularizes upon excess Cu and localizes at the lysosomes and exports Cu by triggering the lysosomal exocytosis pathway (41, 50, 51). Thus, it would be interesting to determine if *Leishmania* Cu-ATPase carries out the dual function of Cu detoxification as well as Cu transport to the secretory pathway as done by its mammalian counterparts, ATP7A and ATP7B.

Recently, it has been demonstrated that *Trypanosome* genome codes for a Cu-ATPase (30). We characterized a putative *Leishmania*-specific P-type ATPase having three predicted N-terminal Cu-binding motifs that share homology with other characterized Cu-ATPases. It is worth mentioning that lower-order eukaryotes carry a single Cu-ATPase; however, with the advent of cellular apicobasal polarity and increasing tissue complexity in higher organisms, this Cu-ATPase branched out into two homologs ATP7A and ATP7B. Our in-depth bioinformatics analysis depicted this putative ATPase as a Cu transporter, which was confirmed with the yeast complementation study. Like its other protozoan counterpart, *Plasmodium berghei* and *Toxoplasma gondii* (52), the LmATP7 is present both in endosomal compartments as well as in plasma membrane. It is well known that mammalian Cu-ATPases, in response to high levels of Cu, relocalize to the vesicles that eventually fuse with the plasma membrane to remove excess Cu from the cell (33, 40). ICP–OES data showing reduced internal Cu level in LmATP7 overexpressed *L. major* suggests the role of LmATP7 as a Cu exporter. Greater survivability of LmATP7-OE strain and reduced survivability of LmATP7-Het strain with respect to Wt promastigotes when challenged with external Cu revealed the importance of LmATP7 in Cu tolerance. Moreover, its expression is regulated by Cu, as observed from the quantitative RT–PCR (qRT–PCR) data indicating its role in the Cu homeostasis mechanism.

To inflict a Cu-induced stress on *Leishmania*, ATP7A of the macrophage traffics to transport Cu to compartments that harbor the parasites. Amastigotes overexpressing LmATP7 exhibit a significantly higher colonization in the macrophages as compared with the Wt control, further establishing the role of LmATP7 in combating host Cu stress. A similar trend of higher parasite survivability of the LmATP7-overexpressing strain was observed upon increasing Cu treatment of the macrophages harboring the amastigotes. It is worth mentioning that for at least up to 200 μM Cu treatment, the parasite burden in LmATP7-OE strain infected macrophages was ≥3 (amastigotes/macrophage cell), which is about twofold higher than the parasite burden in either Wt *Leishmania* or vector control–infected macrophages. Moreover, LmATP7-Het strain exhibited a significantly reduced colonization in the macrophages compared with the Wt control with and without external Cu treatment. Along with reduced survivability, its infectivity also went drastically down. These results, along with repeated failures to generate a complete knockout line for the gene, could suggest its essential nature in maintaining Cu homeostasis and biosynthetic function where it provides Cu to other essential enzymes and proteins.

**Figure 8:**
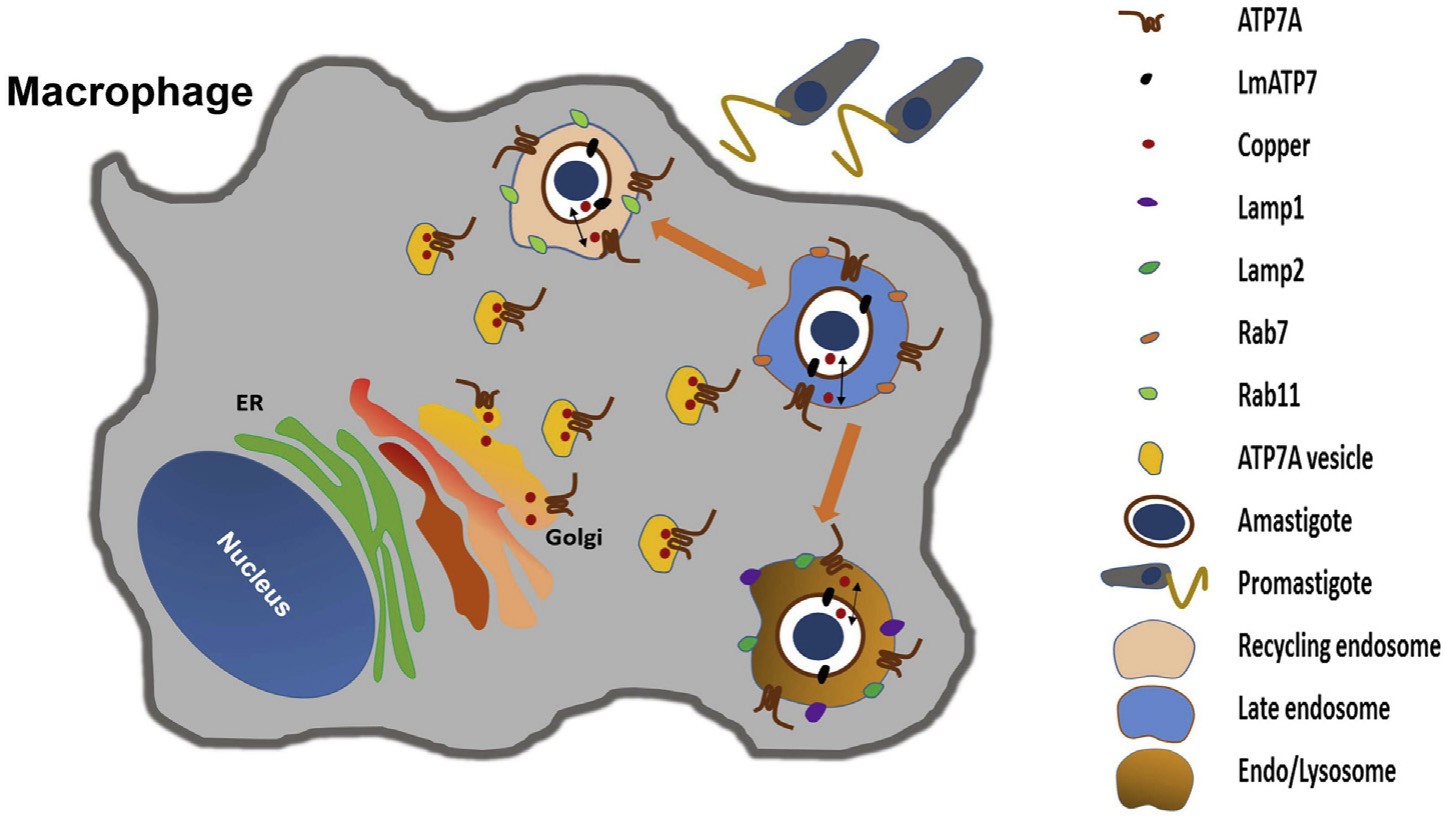
**Proposed model describing the role of parasite and host copper (Cu) P-type ATPases in *Leishmania* infection.** Macrophages channelize Cu *via* ATP7A trafficking from Golgi to the *Leishmania*-positive endo/lysosomal compartments. *Leishmania* amastigotes, in turn, export Cu *via* LmATP7 to reduce the host-mediated Cu stress in order to thrive within the host as indicated by *black double-headed arrows*. *Orange arrows* indicate the endolysosomal pathway followed by amastigote after its entry into the macrophage.

Although it has been reported that several intracellular pathogens try to arrest the phagosome maturation process in the host macrophage cells to avoid transport to lysosomes, *Leishmania* is exceptional since it prefers to reside within the endolysosomal and phagolysosomal compartments (5, 53). There are reports that demonstrate macrophage can actively accumulate and compartmentalize Cu in response to microbial infections (23, 54). In fact, it has been shown that Cu concentration within phagolysosomal compartment of macrophages increases significantly when infected with *Mycobacteria* (22). This might be a crucial step toward parasite killing, since under the acidic environment of the phagolysosome Cu can react with the readily available reactive oxygen and nitrogen species to exert its toxicity (23, 55). Hence, it will be interesting to determine if macrophage cells also utilize Cu in a similar fashion to kill *Leishmania* and the reciprocal response of the parasite. Along this line, our observation of parasite-mediated downregulation of macrophage *ATP7A*, *CTR1*, *CTR2*, and *ATOX1* at early time point, that is, 12 h p.i. with *L. major* implicates the same for the first time. In the putative series of events, we argue that as a host response, the macrophage upregulates ATP7A followed by its Cu transport to the endosomal pathway harboring the parasite. Subsequently, the parasite responds by upregulating the Cu-ATPase, LmATP7, to alleviate the host-induced Cu stress and eventually survives inside the macrophage. Figure 8 illustrates a model based on our findings describing the role of parasite and host Cu P-type ATPases in *Leishmania* infection.

Recently, it has been shown that *Leishmania* hijacks host regulatory pathways that control the expression of a phagolysosomal iron transporter so that it can effectively acquire iron for its survival by increasing the concentration of this metal ion within phagolysosomes (56). However, our study shows that unlike in iron homeostasis, host macrophages try to concentrate Cu within *Leishmania* containing phagolysosomal compartments to kill the pathogen. To counteract the toxic effect, the pathogens have armored themselves with the Cu-ATPase, LmATP7. Hence, it will be extremely important to identify the underlying mechanisms behind the parasite-mediated downregulation of host's Cu utilization proteins as well as how LmATP7 could be targeted to develop novel antileishmanial drug.

## Experimental procedures

Reagents were purchased from Sigma–Aldrich unless mentioned specifically. Primers were obtained from GCC Biotech, and their sequence details are provided in Table S1.

### Plasmids and antibodies

*Leishmania* expression vector (pXG-GFP+) was a generous gift from Dr Stephen M. Beverley (Washington University Medical School).

Following are the antibodies used for experiments: rabbit anti-GFP (catalog no.: BB-AB0065; BioBharati), mouse anti-GP63 (catalog no.: MA1-81830; Invitrogen), rabbit anti-ATP7A (catalog no.: ab125137; Abcam), goat anti-Rab11 (catalog no.: sc-6565; Santa Cruz Biotechnology), mouse anti-Rab7 (catalog no.: sc-376362; Santa Cruz Biotechnology), mouse anti-Lamp1 (Developmental Studies Hybridoma Bank: catalog no.: H4A3), mouse anti-Lamp2 (Developmental Studies Hybridoma Bank: catalog no.: H4B4), donkey anti-rabbit IgG (H + L) Alexa Fluor 488 (catalog no.: A-21206; Invitrogen), donkey antigoat IgG (H + L) Alexa Fluor 568 (catalog no.: A-11057; Invitrogen), and donkey antimouse IgG (H + L) Alexa Fluor 568 (catalog no.: A10037; Invitrogen). FM4-64FX dye (catalog no.: F34653; Invitrogen) was a generous gift from Dr Bidisha Sinha (Indian institute of Science Education and Research [IISER]).

### Parasite and mammalian cell culture

The *L. major* strain 5ASKH was a kind gift from Dr Subrata Adak (Indian Institute of Chemical Biology). *L. major* promastigotes were cultured in M199 medium (Gibco, Thermo Fisher Scientific) supplemented with 15% heat-inactivated fetal bovine serum (Gibco, Thermo Fisher Scientific), 23.5 mM Hepes, 0.2 mM adenine, 150 μg/ml folic acid, 10 μg/ml hemin, 120 U/ml penicillin, 120 μg/ml streptomycin, and 60 μg/ml gentamicin at pH 7.2, and the temperature was maintained at 26 °C. The murine macrophage cell line, J774A.1 (obtained from the National Centre for Cell Science), was grown in Dulbecco's modified Eagle's medium (Gibco, Thermo Fisher Scientific) at pH 7.4 supplemented with 2 mM l-glutamine, 100 U/ml penicillin, 100 μg/ml streptomycin, and 10% heat-inactivated fetal bovine serum at 37 °C in a humidified atmosphere containing 5% CO_2_. Cell number was quantified using a hemocytometer (56).

### Amplification, cloning, and sequencing of *LmATP7*

*LmATP7* was PCR-amplified using gene-specific primer P15/16 from intronless *L. major* genomic DNA (57) and cDNA. Its expression was checked by running the PCR products on 1% agarose gel. Two primer sets P15/16 and P17/18 were used for cloning *LmATP7* into pXG-GFP+ (BamHI and EcoRV sites) and yeast expression vector, p416TEF (as a GFP fusion construct or not) (XbaI and BamHI sites). All three constructs were confirmed by sequencing.

### Transfection

Transfection of DNA into *L. major* was performed using electroporation as described previously (58). Briefly, 10 to 30 μg of DNA construct was resuspended in electroporation buffer (21 mM Hepes, 0.7 mM NaH_2_PO_4_, 137 mM NaCl, and 6 mM glucose; pH 7.4) along with 3.6 × 10^7^ *L. major* promastigotes. The suspension was incubated in a 0.2 cm electroporation cuvette for 10 min on ice, following which electroporation was performed on the Bio-Rad Gene Pulsar at 450 V, 550 μF capacitance. Transfected cells were selected in appropriate antibiotic-containing medium.

### Generation of *L. major* strain expressing GFP-tagged *LmATP7*

*LmATP7-GFP* construct or vector control (only pXG-GFP) was transfected into Wt *L. major* promastigotes by electroporation as described previously in the transfection section to generate the overexpressing *LmATP7* strain or only GFP-expressing strain (59).

### Targeted replacement of *LmATP7* allele of *L. major* with antibiotic-selectable markers

To replace an allele of LmATP7 with NEO gene cassette, we generated the deletion construct in pXG-NEO vector, as described previously (58). Briefly, 741 bp from the 5′ flanking region (starting 174 bp upstream of the start codon) and 740 bp from the 3′ flanking region of the *LmATP7* open reading frame were PCR-amplified from *L. major* genomic DNA using primer sets P19/P20 and P21/P22, respectively. HindIII and SalI-digested 5′ flanking region and SmaI and EcoRI-digested 3′ flanking region of LmATP7 were ligated on the NEO-encoding open reading frame in pXGNEO plasmid, respectively. The construct was verified by sequencing, following which they were digested with HindIII and BamHI to generate the linearized targeting cassette: 5′LmATP7-NEO-LmATP73′. To generate LmATP7 +/− strain, Wt *L. major* promastigotes were transfected with 10 μg of 5′ LmATP7-NEO-LmATP73′ linearized cassette. LmATP7 +/− heterozygous strain was selected and maintained in the presence of 100 μg/ml G418 sulphate.

### Effect of external Cu on promastigote growth kinetics

To assess the effect of Cu on the growth of *Leishmania* parasites, promastigotes were cultured in M199 medium as mentioned earlier in the presence of cupric chloride at a final concentration of 0, 50, 100, 200, 300, or 500 μM. Four different strains of *L. major*, including Wt, vector control, LmATP7-OE, and LmATP7-Het, were used during this study. Parasites were quantified by hemocytometer-based counting using trypan blue after 24, 48, and 72 h postincubation in the presence of Cu to evaluate the growth kinetics. Similarly, to check if Cu exerts any toxic effect over macrophage growth, J774A.1 macrophage cultured in Dulbecco's modified Eagle’s medium was supplemented with cupric chloride at a final concentration of 0, 50, 100, 200, 300, or 500 μM. Cell number was measured using hemocytometer following trypan blue staining at 24 and 48 h postincubation. Each of the aforementioned studies was performed in triplicate to reach a meaningful statistical analysis.

### Infection of macrophages with *L. major* and estimation of intracellular parasite burden

Infection of J774A.1 murine macrophages with late log-phase *L. major* promastigotes of either Wt, vector control, LmATP7-OE, or LmATP7-Het was performed as described previously at a parasite-to-macrophage ratio of 30:1 (56). Briefly, J774A.1 macrophage cells were incubated with *L. major* promastigotes for 12 h following which the nonphagocytosed parasites were removed and the infection was allowed to continue for 12, 24, 30, or 48 h. After 12 and 30 h time points p.i., cells were harvested to carry out qRT–PCR. After 24 and 48 h time points p.i., cells were washed and fixed with acetone–methanol (1:1). Antifade mounting medium containing 4′,6-diamidino-2-phenylindole (DAPI) (VectaShield from Vector Laboratories) was used to stain the nuclei of the fixed infected macrophages. Intracellular parasite burden represented as amastigotes/100 macrophage cell was quantified by counting the total number of DAPI-stained nuclei of macrophages and *L. major* amastigotes in a field (at least 100 macrophages were counted from triplicate experiments). For measuring the amastigote/macrophage counts under different concentrations of Cu treatment, a similar experiment was performed where Cu was added post 12 h of initial incubation of macrophages with the parasites.

### qRT–PCR of macrophage- and *Leishmania* strain–specific Cu regulators

Total RNA was isolated from uninfected macrophages, Wt *L. major*–infected macrophages as well as from Wt, LmATP7-OE, and LmATP7-Het *L. major* strains using TRIzol reagent (Invitrogen). DNA contamination was removed with DNaseI (Invitrogen) treatment. Verso cDNA synthesis kit (Thermo Fisher Scientific) was used for cDNA preparation from 1 μg of total RNA. Following primers were used for quantification of transcript level of different genes: P1/P2 (*ATP7A*), P3/P4 (*CTR1*), P5/P6 (*CTR2*), P7/P8 (*ATOX1*), P9/P10 (*LmATP7*), P23/24 (*GFP-LmATP7*), and P25/26 (*kDNA*). Real-time PCR was performed with SYBR green fluorophore (Bio-Rad) using 7500 real-time PCR system of Applied Biosystems. The relative transcript level of macrophage-specific genes was normalized using Wt cells as the reference sample and the *β-actin* gene as an endogenous control. In case of LmATP7 mRNA expression, *rRNA45* gene was taken as endogenous control. Amplification of *β-actin* from macrophage and *rRNA45* from *Leishmania* cells was performed using the primer sets P11/P12 and P13/14, respectively. The experiments were performed as per minimum information for publication of quantitative real-time PCR experiments guidelines.

### Immunofluorescence studies and image analysis

Macrophages were seeded at a density of ∼2 × 10^5^ cells on glass coverslips, and following different experimental set ups to required time points, cells were fixed using acetone:methanol (1:1) for 10 min. Cells were then washed with 1× PBS followed by permeabilization using 0.1% Triton-X 100. Both fixation and permeabilization were carried out by keeping it on ice. Cells were then again washed with ice-cold 1× PBS and blocked with 0.2% gelatin for 5 min at room temperature. Incubation with primary antibodies (anti-ATP7A 1:200; anti-Rab11 1:200; anti-Rab7 1:200; anti-Lamp1 1:50; and anti-Lamp2 1:50) was performed for 2 h at room temperature followed by 1× PBS wash. Cells were reincubated for 1.5 h at room temperature with either of the following secondary antibodies, goat anti-rabbit Alexa Fluor 488 (1:1000) for ATP7A, donkey antigoat Alexa Fluor 564 (1:1000) for Rab11, goat antimouse Alexa Fluor 568 (1:1000) for Rab7, Lamp1, and Lamp2. Following two 1× PBS washes, coverslips were mounted on glass slides using Fluoroshield with DAPI mountant (catalog no.: F6057; Sigma–Aldrich). All images were visualized with Leica SP8 confocal platform using oil immersion 63× objective and were deconvoluted using Leica Lightning software.

To determine LmATP7 localization in *L. major*, Wt or GFP-only or LmATP7-GFP-expressing cells were mounted on poly l-lysine–coated coverslips, fixed with acetone:methanol (1:1), and permeabilized with 0.1% Triton X-100 at 4 °C. 0.2% gelatine was used to block nonspecific binding. Cells were then incubated with either anti-GFP primary antibody or anti-GP63 antibody (1:200) for 1.5 h. Thereafter, cells were washed with ice-cold PBS and incubated with either goat anti-rabbit Alexa Fluor 488 secondary antibody (1:800) for GFP or with goat antimouse Alexa Fluor 568 secondary antibody (1:600) for GP63 for 1.5 h in the dark. After incubation, cells were washed with PBS and embedded in antifade mounting medium containing DAPI. For FM4-64FX staining, LmATP7-GFP expressing cells (under basal or 2 h of 50 μM Cu treatment) were incubated for 1 min or 10 min with the dye prior to the fixation step. Immunostaining was performed using the aforementioned protocol with primary antibody, anti-GFP (1:200) and secondary antibody, and goat anti-rabbit Alexa Fluor Plus 488 (1:800). To check ATP7A crossreactivity, Wt *L. major* cells were similarly immunostained with primary antibody, anti-ATP7A (1:200) and secondary antibody, and goat anti-rabbit Alexa Fluor Plus 488 (1:800). All images were visualized with Leica SP8 confocal platform using oil immersion 63× objective and were deconvoluted using Leica Lightning software.

### Bioinformatics analysis of LmATP7

The gene (*LmATP7*) sequence for putative Cu-transporting ATPase-like protein was acquired from TriTrypDB (60). Clustal Omega was used to generate multiple sequence alignment (61). Multiple sequence alignment visualization, analysis, and editing were performed using Jalview program (Geoff Barton's Group at the University of Dundee) (62).

### Homology modeling and structural validation

We modeled the three HM-binding domains of *Leishmania* P-type Cu-ATPase transporter. The sequence of the three domains that are used for homology modeling is as follows:

HM1: TLNVFGTTCRGCAQHVQENLMTLEGVHSVSVDLDAQLAEVDVDATDAATEFRIEKKMVSMGY

HM2: LLIEGMSCTSCAARIEAKLKQLKGVLGASVNFSAMSGQVLHNPALAPLPKVVSCVADMS

HM3: DGEQPQEGECKCPTNLQAVPVHVGSVMSGYEHRLVVLGMSCASCAARIEHRLRQMPTVLNCTVSFVTGTAV

All these three contain the putative Cu-binding motif (CXXC), which is thought to be responsible for Cu transport. In order to provide a possible structure, we performed homology modeling followed by molecular dynamics simulations. There is no experimentally resolved 3D structure for the metal-binding domains of *Leishmania* ATP7. In order to find the most similar sequence whose structure is known (template) to each of the target sequence, sequence similarity search was performed on the BLASTp online server (http://blast.ncbi.nlm.nih.gov), which were subsequently used as templates for homology modeling performed using the Modeller 9.25 program (by Andrej Šali) (63). This yielded the initial structural guesses for the HMs. The best model was selected with regard to the best Discrete Optimization Protein Energy score (64). Initially, each of the aforementioned best models was solvated by ∼40,000 TIP3P water molecules in a box of dimension 80 × 80 × 80 Å3 (65). The physiological concentration (150 mM) of Na^+^ and Cl^−^ ions along with extra ions was used to neutralize the system. These were subsequently energy minimized using the steepest descent method for 10,000 steps, followed by heating it to 300 K in 200 ps using Berendsen thermostat and barostat with coupling constant of 0.6 ps. Restraints of 25 kcal/mol/Å2 were applied on heavy atoms during the heating process. Thereafter, equilibration was carried out for 2 ns at constant temperature (300 K) and pressure (1 bar) without any restraints using the same thermostat and barostat with coupling constants of 0.2 ps each. The last 100 ps of NPT simulation was used to calculate the average volume the same, which was used in the final 1000 ns unrestrained NPT equilibration using the velocity-rescale thermostat (66) and a Parrinello–Rahman barostat (67) with coupling constant of 0.2 ps. During the simulation, LINCS algorithm was used to constrain all the bonds, and particle-mesh Ewald method (68) was used for electrostatics. The distance cutoffs for the van der Waals and electrostatic long-range interaction were kept at 10 Å. The time step for each simulation was taken to be 2 fs. All the simulations were performed using molecular dynamics software GROMACS 2019.4 (University of Groningen Royal Institute of Technology Uppsala University) (69) with parameters from the AMBER99SB force field (70).

### Determination of cellular Cu concentration by ICP–OES

Wt, LmATP7-GFP overexpressing, and LmATP7-Het *L. major* cells were pelleted down and washed with ice-cold 1× Dulbecco's PBS (DPBS) (catalog no.: 14200075; Gibco). Repeated washing was performed (five times) to ensure no trace of Cu outside the cells. The pellets were dissolved in DPBS and counted by hemocytometer. About 1 × 10^6^ cells were digested overnight with 120 μl of 65% suprapur HNO_3_ at 95 °C. After digestion, samples were diluted in 6 ml of 1× DPBS and syringe filtered through a 0.22 micron filter. Cu calibration was done by acid digestion of Cu foil (procured from Alfa Aesar) in 10 ml suprapur HNO_3_ for 1 h (Microwave Diathermy or MWD conditions: power = 400 W; temperature = 1000 °C; and hold time= 1 h). From the obtained solution, solutions of varying Cu strengths were prepared and used for calibration. Cu concentrations of the samples and acid-digested DPBS (blank) were determined using a Thermo Scientific ICP–OES iCAP 6500.

### Strains, media, and growth conditions for yeast complementation assay

For yeast complementation studies, *S. cerevisiae* BY4742 (Wt) strain (*MATα his3Δ1 leu2Δ0 lys2Δ0 ura3Δ0*) and an *S. cerevisiae* strain carrying a *CCC2* deletion (ccc2Δ) in the BY4742 background purchased from Euroscarf were used. Yeast extract, peptone, and dextrose medium was used for routinely maintaining both Wt and deletion strains. For complementation assay, synthetic defined (SD) minimal media containing yeast nitrogen base, ammonium sulfate, and dextrose supplemented with histidine, leucine, lysine, and methionine (80 mg/l) were used. Yeast transformations were carried out using lithium acetate method (71). Human *ATP7B* mRNA was cloned in p416TEF vector (as a positive control) and confirmed by sequencing. *LmATP7*, *GFP-LmATP7*, and human *ATP7B* constructs along with empty vector p416TEF were used to transform Wt and ccc2Δ strains. Yeast transformants were selected and maintained on SD medium without uracil (SD-Ura) at 30 °C. p416TEF vector contains URA3 selection marker allowing growth in the absence of uracil. Wt strain was transformed with empty vector to allow its growth on SD-Ura.

### *In vivo* functional complementation assay in *S. cerevisiae* by dilution spotting

Yeast transformants were grown overnight at 30 °C with shaking at 200 rpm in SD-Ura medium. Primary culture was used to inoculate secondary culture in the same selective medium and allowed to grow at 30 °C till an absorbance reached about 0.6 at 600 nm. The cells were centrifuged, washed, and diluted in sterile water at an absorbance of 0.2 at 600 nm. Serial dilutions were then made with sterile water (absorbance at 600 nm = 0.2, 0.02, 0.002, and 0.0002), and 10 μl of cell suspension from each were spotted on plates containing SD-Ura medium with or without 250 μM iron chelator, Ferrozine (Sisco Research Laboratories). Plates were incubated at 30 °C for 3 days, and photographs were taken.

### Mice infection and determination of lesion size and parasite load

Six- to eight-week-old female BALB/c mice were obtained from the National Institute of Nutrition and housed in our institutional animal facility. The animal studies involving mice were approved by IISER Kolkata Animal Ethics Review Board. Experiments with these mice were conducted according to the Committee for the Purpose of Control and Supervision of Experiments on Animals guidelines and Institutional Animal Ethics Committee–approved protocol. Wt, vector control, LmATP7-OE, or LmATP7-HET *L. major* strains were grown as described earlier in this section. For infection, 1 × 10^6^ stationary phase promastigotes of these four *Leishmania* strains were suspended into PBS and injected into left hind footpad. The development and progression of footpad lesion was monitored weekly postinfection by blindly measuring the left hind footpad with respect to the uninfected right hind footpads with calliper (42). At week 10 and 14 postinfection, mice were sacrificed, and parasite burden in the footpads of infected mice was quantified by limiting dilution analysis as previously reported (72, 73).

### Image analysis

Images were analyzed using image analysis software, ImageJ (by Wayne Rasband) (74). Colocalization_Finder plugin was used for colocalization studies. Regions of interests were drawn manually on the best z-stack for each cell. Manders' colocalization coefficient (75) was measured using macro codes for quantifying colocalization (76).

### Statistical analysis

For statistical analysis and plotting, ggplot2 package was used in R, v-4.1.1 (77, 78). Statistical analyses were performed by Student's *t* test or by Wilcoxon rank-sum test.

## Data availability

All data described in this article are contained within the article.

## Supporting information

This article contains supporting information.

## Conflict of interest

The authors declare that they have no conflicts of interest with the contents of this article.
