# Supplementary material for: A novel leishmanial copper P-type ATPase plays a vital role in parasite infection and intracellular survival
Source: J Biol Chem. 2021 Dec 25;298(2):101539. doi: 10.1016/j.jbc.2021.101539 (PMC8800121; doi:10.1016/j.jbc.2021.101539)
Supplement: Supplemental Table S1 [file mmc1.docx]

Table S1. Oligonucleotides used in this paper

| Primer | Primer Sequence (5’- 3’) |
| --- | --- |
| P1 | GAAGAGGTCGGACTGCTGTC |
| P2 | CCTTAGTAATGCCAACCTGAGAAGC |
| P3 | CAAGATAGCCCGAGAGGGTC |
| P4 | GATGTGCAGCACTGTCTGC |
| P5 | AGATGAGGCCGTGCTTCTC |
| P6 | CCTGGTCTGGTCCCAAGATG |
| P7 | GCACGAGTTCTCCGTGGAC |
| P8 | GGGCCAAGGTAGGAAACAGC |
| P9 | TGTCATCCGGTATCTGCACA |
| P10 | CGGCGTACACATCTTTCGAG |
| P11 | GGCTGTATTCCCCTCCATCG |
| P12 | CCAGTTGGTAACAATGCCATG |
| P13 | CCTACCATGCCGTGTCCTTCTA |
| P14 | AACGACCCCTGCAGCAATAC |
| P15 | CGCGGATCCATGGGCGCGACGACT |
| P16 | CGAGATATCTGACATGAAATAGAAATCGCGTT |
| P17 | CGCGGATCCATGGGCGCGACGACTGC |
| P18 | CCCAAGCTTTCACATGAAATAGAAATC |
| P19 | AAGCTTACGATTCGTACCGCGACG |
| P20 | GTCGACAAGCAGACGCGTCTCCTC |
| P21 | CCCGGGCTGCCTCCGCGCACATTG |
| P22 | GGATCCCGTTGCACGAAGCAAAAG |
| P23 | GAGCAAAGACCCCAACGAGA |
| P24 | AACGCTCACACGAGGATCTG |
| P25 | AAGGGTGAACGCCAAAAACG |
| P26 | GTTCGGTTAATCCGCGAACG |
